# A survey of the representativeness and usefulness of wastewater-based surveillance systems in 10 countries across Europe in 2023

**DOI:** 10.2807/1560-7917.ES.2024.29.33.2400096

**Published:** 2024-08-15

**Authors:** Guido Benedetti, Lene Wulff Krogsgaard, Sabine Maritschnik, Hans Peter Stüger, Veronik Hutse, Raphael Janssens, Soile Blomqvist, Tarja Pitkänen, Anastasia Koutsolioutsou, Eszter Róka, Marta Vargha, Giuseppina La Rosa, Elisabetta Suffredini, Henry-Michel Cauchie, Leslie Ogorzaly, Rudolf FHJ van der Beek, Willemijn J Lodder, Elisabeth Henie Madslien, Jose Antonio Baz Lomba, Steen Ethelberg

**Affiliations:** 1 Department of Infectious Disease Epidemiology and Prevention, Statens Serum Institut, Copenhagen, Denmark; 2 Austrian Agency for Health and Food Safety (AGES), Institute for Surveillance and Infectious Disease Epidemiology, Vienna, Austria; 3 Austrian Agency for Health and Food Safety (AGES), Department of Statistics and Analytical Epidemiology, Vienna, Austria; 4 COVID-19 Wastewater Surveillance, Epidemiology of Infectious Diseases, Epidemiology and Public Health, Sciensano, Brussels, Belgium; 5 Expert Microbiology Unit, Finnish Institute for Health and Welfare, Helsinki, Finland; 6 Expert Microbiology Unit, Finnish Institute for Health and Welfare, Kuopio, Finland; 7 Department of Food Hygiene and Environmental Health, Faculty of Veterinary Medicine, University of Helsinki, Helsinki, Finland; 8 Department of Environmental Health and Monitoring of Smoking Cessation, Directorate of Epidemiology and Prevention of Non-Communicable Diseases and Injuries, National Public Health Organisation, Marousi, Greece; 9 National Center for Public Health and Pharmacy, Budapest, Hungary; 10 National Center for Water Safety (CeNSia), Istituto Superiore di Sanità, Rome, Italy; 11 Department of Food Safety, Nutrition and Veterinary Public Health, Istituto Superiore di Sanità, Rome, Italy; 12 Luxembourg Institute of Science and Technology (LIST), Environmental Research and Innovation Department (ERIN), Belvaux, Luxembourg; 13 Centre for Infectious Disease Control, National Institute for Public Health and the Environment, Bilthoven, the Netherlands; 14 Department of Infection Control and Preparedness, Norwegian Institute of Public Health, Oslo, Norway; 15 Department of Public Health, Global Health Section, University of Copenhagen, Copenhagen, Denmark

**Keywords:** Wastewater-based epidemiological monitoring, Public Health Surveillance, Representativeness, Usefulness, Europe, SARS-CoV-2, infectious disease

## Abstract

Wastewater-based surveillance (WBS) has become a widespread method to monitor transmission of SARS-CoV-2 and other human pathogens in Europe. We conducted a survey about WBS systems’ objectives, approaches, representativeness and usefulness in 10 invited European countries in 2023, i.e. Austria, Belgium, Denmark, Finland, Greece, Hungary, Italy, Luxembourg, the Netherlands and Norway. All countries completed the study questionnaire about their SARS-CoV-2 WBS systems, and shared information about WBS of other pathogens as deemed relevant. SARS-CoV-2 WBS systems primarily monitored national and subnational trends (population coverage: 25–99%), and a majority (8/10) also tracked variant distribution. Nine of 10 countries reported that their SARS-CoV-2 WBS systems were representative of their population and all countries remarked that the findings were valuable for public health decision-making. Results were shared with relevant public health authorities and published via dedicated websites and/or dashboards. WBS systems of other pathogens were mostly in the early stages, with some countries implementing pilots. Notable exceptions were the well-established poliovirus surveillance systems in Finland, Italy and the Netherlands. This study brings understanding the diverse landscape of WBS in Europe, offering insights for future developments and collaborations. Furthermore, it highlights the need for further integration of WBS into other European surveillance systems.

## Introduction

Wastewater-based surveillance (WBS) has been successfully used for decades for public health purposes in Europe, e.g. to monitor the circulation of poliovirus in the population [1]. Although not a new tool, it was during the severe acute respiratory syndrome coronavirus 2 (SARS-CoV-2) pandemic that WBS was incorporated alongside other, more traditional surveillance tools such as community and clinical testing [2-4].

During the SARS-CoV-2 pandemic, the European Commission recommended that all European Union (EU) countries implement SARS-CoV-2 WBS beginning in March 2021 and no later than October 2021 [5]. This initiative was strengthened in 2022 by the World Health Organization interim guidance *Environmental surveillance for SARS-COV-2 to complement public health surveillance* [6]. Since then, several European countries have developed WBS systems to monitor the occurrence and spread of SARS-CoV-2, as documented by the Digital European Exchange Platform of the EU Sewage Sentinel System for SARS-CoV-2 [7,8]. By 2023, the pressure of the SARS-CoV-2 pandemic on communities and their health systems had notably diminished and individual testing was generally at its lowest level, yet WBS has remained at the frontline of SARS-CoV-2 surveillance.

Disease surveillance is information that can and should be used for action and the knowledge gathered can be translated into intervention through decision-making [9-11]. Asking questions like ‘Is WBS beyond SARS-CoV-2 relevant in Europe?’ and ‘What are the problems to be solved next and through what strategies?’ become key to address preparedness against viral and bacterial pathogens with a pandemic potential. Furthermore, the 2022 revision of the EU’s Urban Wastewater Treatment Directive aimed to ‘require EU countries to monitor pathogens in wastewater’ [12,13]. It is therefore important to review how the WBS experience gained with the SARS-CoV-2 pandemic is being capitalised upon and applied to prevent current and future threats. Through a survey conducted at the end of 2023, we collected and critically described knowledge about the objectives, approaches, representativeness and usefulness of WBS systems of pathogens of human relevance in 10 European countries. Moreover, we discussed the experiences, lessons learned and future opportunities/perspectives from the participating European countries.

## Methods

### Study design and setting

This was a narrative survey, via an open-question questionnaire, about the objectives, approaches, representativeness and usefulness of WBS systems of pathogens of human relevance as they were functioning during 2023 in 10 invited European countries.

This study was designed and coordinated by researchers at Statens Serum Institut (SSI), Denmark. Ten countries were identified during summer 2023 based on their documented experience with WBS and their active participation in designing the Joint Action EU-WISH (European Union Wastewater Integrated Surveillance for Public Health) project of the EU4Health programme [14,15], i.e. Austria, Belgium, Denmark, Finland, Greece, Hungary, Italy, Luxembourg, the Netherlands and Norway. Researchers and public health officers from the 10 countries who were directly involved in the management of their respective national WBS systems and were committing to the EU-WISH project (henceforth defined as ‘participants’) received an invitation letter (September 2023), which explained the purpose of this study and the study questionnaire. All participants returned their answers by November 2023.

### Study questionnaire

Participants were invited to answer the questionnaire in regard to any human pathogenic microorganisms of relevance in their respective country and based on their knowledge and experience. Although SARS-CoV-2 was known to be monitored by all countries, no requirements were made concerning the pathogens to be included in the survey. Other WBS targets, e.g. contaminants, were not covered by this study. The questionnaire was primarily based on *Data quality monitoring and surveillance system evaluation – A handbook of methods and applications*, published by the European Centre for Disease Prevention and Control (ECDC) [16]. 

The representativeness of the surveillance systems was investigated as the capacity to ‘accurately describe[s] the occurrence of a health-related event over time and its distribution in the population’, focusing on the coverage of the system. The usefulness of the systems was determined as the use made of their respective ‘surveillance results […] for public health action’ [16]. We defined the beneficiaries of the surveillance activities as the population of the country. Table 1 shows the topics of the questionnaire. Two researchers at SSI (GB and LWK) independently reviewed and pre-tested the questionnaire by answering its questions with information from the Danish SARS-CoV-2 WBS system.

**Table 1:** Overview of the topics of the questionnaire on wastewater-based surveillance in 10 European countries, 2023

Information about the WBS system(s)
Name of the system(s)
Relevant webpage
Public, available documentation/information/literature about the WBS system(s), with links
Approach of the WBS system(s)
Pathogen(s) under surveillance
Brief description of the system(s), including the number of sampling sites, number of weekly samples, sampling methods and number of laboratories performing the analyses. Can also include information about e.g. sentinel surveillance, seasonal surveillance, ad hoc setup for emerging threats.
Objectives of the system(s)
Use of non-wastewater data in the WBS system(s)
Sectors and actors implementing WBS (including their role in funding)
Use case – if available, describe and/or reference how the implementers of the system(s) perform tasks (list of actions) and how they use the information generated by the system(s).
Representativeness – ‘A public health surveillance system that is representative accurately describes the occurrence of a health-related event over time and its distribution in the population by place and person’ [16]
Population under surveillance: what is it and how is it identified?
Describe and quantify the geographical coverage of the system(s), e.g. regional/national; rural/urban.
Describe and quantify the frequency of the data collection.
Describe relevant infrastructural, legislative, financial or other matters that determine the coverage of the system(s).
Describe major changes in the representativeness of the system(s) since 2020.
Considering the most relevant pathogen under wastewater surveillance today, how do you consider the system(s) to be representative of the population residing in the country?
Usefulness – ‘Usefulness implies that surveillance results are used for public health action. Assessing usefulness consists in taking inventory of actions that have been taken in conjunction with the surveillance system’ [16]
Describe how the results of the system(s) are communicated (internally/externally).
Describe how the information gathered by the system(s) is used and for what purpose, e.g. detection of pathogens or other hazards; estimation of disease burden; detection of outbreaks; description of disease distribution, spread, trends, modality, risk factors; hypotheses to stimulate research; measuring results of control measures; guidance for public health planning.
Describe the actors involved in using the information gathered by the system(s).
Provide example(s) of how public health actions are based on the information gathered by the system(s).
Describe how the actors implementing WBS monitor and how/if the surveillance system(s) is used for public health actions.
Considering the most relevant pathogen under wastewater surveillance today, how do you consider the system(s) to be useful to make decisions of public health relevance?
Role of the community (beneficiaries)
Describe the role of the community (beneficiaries of the surveillance activities) in defining the objectives, designing the representativeness and assessing the usefulness of the WBS system(s) today.

WBS: wastewater-based surveillance.

The questionnaire specifically enquired about the functioning of WBS systems in 2023. Supplementary Materials include a full overview of the study questionnaire.

### Data analysis

Answers to the questionnaire were received by SSI researchers and assessed for their completeness. Two researchers (GB and LWK) independently reviewed answers and recorded similarities and differences across the different national WBS systems. Noteworthy experiences were also highlighted. Pathogen-specific WBS system characteristics were also extracted from answers, including the objectives of the system, the population and geographical coverage, the number of sampling sites, the frequency of sampling and the technical process of sampling. Two online meetings were organised with all participants to present and discuss the received answers, to define the analysis plan and to discuss findings.

## Results

**Figure 1:**
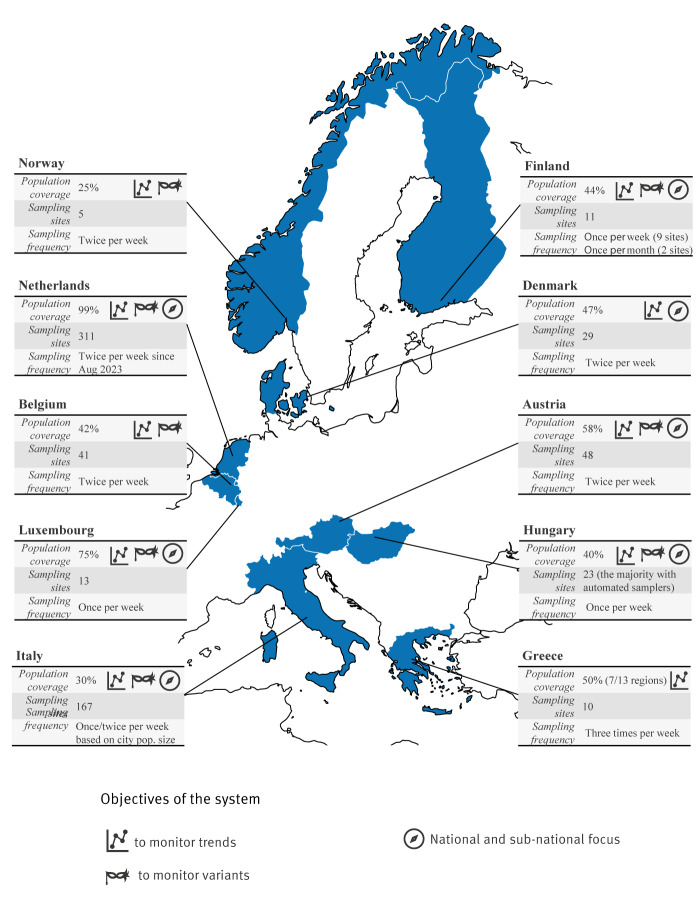
Overview of the SARS-CoV-2 wastewater-based surveillance systems in 10 European countries, 2023

**Figure 2:**
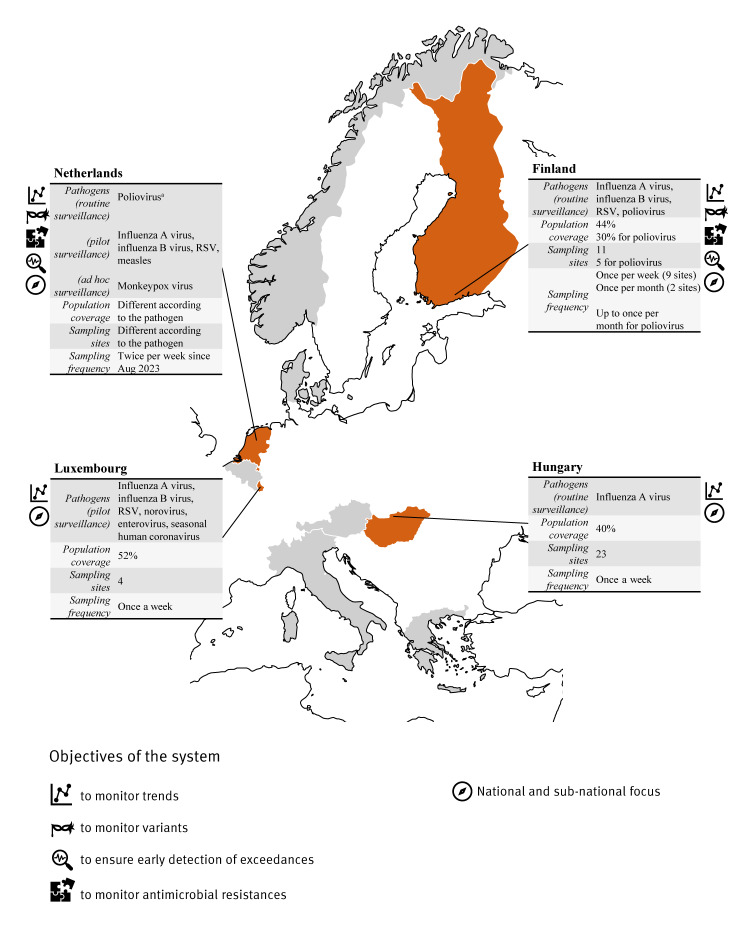
Overview of the wastewater-based surveillance systems targeting pathogens other than SARS-CoV-2 in 10 European countries, 2023

RSV: respiratory syncytial virus; SARS-CoV-2: severe acute respiratory syndrome coronavirus 2.

^a^ Grab samples instead of composite sample.

All 10 invited countries accepted to participate and answered the study questionnaire about their WBS systems, i.e. Austria, Belgium, Denmark, Finland, Greece, Hungary, Italy, Luxembourg, the Netherlands and Norway. All questionnaires were complete with regard to WBS of SARS-CoV-2, while information was shared for other pathogens as the countries deemed relevant. Participants either used one questionnaire to report about a single pathogen or merged the answers of more pathogens into one questionnaire. The survey responses for all countries are provided as Supplementary Materials. An overview of the included WBS systems for SARS-CoV-2 (Figure 1) and other pathogens (Figure 2) illustrates the population coverage, the number of sampling sites, the sampling frequency, the sampling method and objectives. 

SARS-CoV-2: severe acute respiratory syndrome coronavirus 2.

All systems use 24 h composite samples. Samples are taken as either flow-proportional or time-proportional composite samples. Unless specified otherwise, the sampling frequency did not change during 2023.

The webpage of the Digital European Exchange Platform (EU4S-DEEP) [7] provides a rich collection of information about the wastewater infrastructure of European countries, including the operating treatment plants.

All systems use 24 h composite samples. Samples are taken as either flow-proportional or time-proportional composite samples. Unless specified otherwise, the sampling frequency did not change during 2023.

Countries (e.g. Italy) reported the presence of poliovirus WBS, although a description of the system was not reported in the questionnaire. This overview is therefore only partially representative of the pathogens other than SARS-CoV-2 under WBS in the ten participating countries.

Generally, SARS-CoV-2 WBS had the primary objective to monitor national and sub-national trends across countries, with a majority of systems (8/10) also monitoring the distribution of variants. Generally, countries relied on a network of nationally distributed laboratories. Concerning SARS-CoV-2, all countries reported to integrate clinical surveillance data, e.g. case numbers, hospitalisations and vaccination coverage, in their WBS systems. International data from variants of concern were also considered for the sake of comparison. However, no country reported to routinely monitor publicly available data about SARS-CoV-2 trends from abroad, e.g. from neighbouring countries. Nine systems were publicly funded (with nine countries reporting about funding), with a diverse network of actors involved, given the country-specific setup, e.g. local/municipal health authorities were involved in Belgium, Hungary and Norway, while universities were in Austria, Belgium and Greece. Wastewater treatment plants also had a variety of participation and funding mechanisms. Supplementary Materials report the received answers to the questionnaire. Table 2 summarises the relevant webpages and visualisation dashboards from the analysed WBS systems.

**Table 2:** Overview of the relevant wastewater-based surveillance webpages and visualisation dashboards in 10 European countries, 2023

Country	Relevant webpage(s)/dashboard(s)	Link(s)
Austria	Austrian SARS-CoV-2 wastewater-based surveillance system	https://abwassermonitoring.at
Belgium	Wastewater surveillance Covid-19	https://wastewater.sciensano.be/dashboard/covid19/en/
	CoVWWSurv - National surveillance of SARS-CoV-2 and its variants in wastewater	https://www.sciensano.be/en/projects/national-surveillance-sars-cov-2-and-its-variants-sewage
Denmark	The Danish SARS-CoV-2 wastewater-based surveillance system	https://en.ssi.dk/covid-19/national-surveillance-of-sars-cov-2-in-wastewater
Finland	Wastewater-based surveillance of respiratory viruses and AMR in Finland	https://thl.fi/en/web/infectious-diseases-and-vaccinations/surveillance-and-registers/wastewater-monitoring
	Environmental surveillance for poliovirus in Finland	https://thl.fi/fi/web/infektiotaudit-ja-rokotukset/taudit-ja-torjunta/taudit-ja-taudinaiheuttajat-a-o/polio/polion-jatevesiseuranta
Greece	National Wastewater Surveillance System	https://eody.gov.gr/epidimiologika-statistika-dedomena/evdomadiaies-ektheseis/evdomadiaies-ektheseis-epidimiologikis-epitirisis-anapneystikon-loimoxeon
Hungary	National Wastewater-based Surveillance System	https://www.nnk.gov.hu/index.php/koronavirus/szennyvizvizsgalatok
Italy	The Italian SARS-CoV-2 wastewater-based surveillance system	https://www.iss.it/cov19-acque-reflue
Luxembourg	CORONASTEP	https://www.list.lu/en/covid-19/coronastep
Netherlands	The Dutch National Wastewater Surveillance programme Open datasets	https://data.rivm.nl/meta/srv/dut/catalog.search#/metadata/a2960b68-9d3f-4dc3-9485-600570cd52b9
	SARS-CoV-2 – Current national information	https://www.rivm.nl/en/coronavirus-covid-19/current/weekly-update
	Dutch National Wastewater Surveillance programme	https://www.rivm.nl/en/sewage-research/covid-19
	Poliovirus research	https://www.rivm.nl/en/sewage-research/polio
	Antimicrobial resistance research	https://www.rivm.nl/en/antimicrobial-resistance
Norway	The Norwegian SARS-CoV-2 wastewater surveillance system	https://www.fhi.no/en/in/surveillance/wastewater-surveillance-of-infectious-diseases/results-from-wastewater-surveillance

AMR: antimicrobial resistance; SARS-CoV-2: severe acute respiratory syndrome coronavirus 2.

### Representativeness

The median population coverage for SARS-CoV-2 WBS systems was 46% of the national population, ranging from 25% in Norway to 99% in the Netherlands (Figure 1). With the exception of Hungary and the Netherlands, countries documented a geographical downscaling of their SARS-CoV-2 WBS systems occurring in 2023 in comparison to the period 2020–22. The majority of countries (9/10) reported that their SARS-CoV-2 WBS systems were representative of their population, whereas Norway reported the system to be only partially representative, with samples primarily taken from the largest cities in the south of the country. In most countries (7/10), the coverage was measured by the administrative level of regions (Nomenclature of Territorial Units for Statistics 2, NUTS-2) [17].

Wastewater-based surveillance primarily targeted larger urban and high-density populated areas, particularly in Austria, Greece, Italy and Norway. Generally (6/10 countries), the sampling frequency for SARS-CoV-2 analysis was twice weekly, ranging 1–3 times weekly. Countries also reported the capacity to scale the frequency up based on needs, i.e. Belgium, Denmark, Finland, Greece, Hungary and Luxembourg.

The SARS-CoV-2 target population, i.e. the share of the country population covered by the wastewater treatment plants included in the WBS system, was identified with various methods. In Denmark, a R Shiny application was developed to identify sampling sites for SARS-CoV-2 based on population figures and operational goals. Similarly, analysis models were designed in Belgium to ensure the representativeness of the system. Luxembourg reported a challenge in defining the relevant target population, given the large share of commuters from abroad during working days. Countries reported to have evaluated the desired outcomes of their respective systems in view of costs to define their coverage, i.e. Belgium, Hungary and the Netherlands.

Either as pilot or routine surveillance, Hungary, Finland, Luxembourg and the Netherlands reported including additional pathogens into their monitoring, e.g. influenza A virus, influenza B virus, respiratory syncytial virus, measles virus, extended spectrum beta-lactamase-producing *Escherichia coli* and poliovirus (see Figure 2). The Netherlands and Finland reported a different population coverage according to the pathogen under surveillance and the epidemiological situation. Sampling frequency was 1–2 times weekly. The population coverage for pathogens other than SARS-CoV-2 ranged from 40% in Hungary to 44% in Finland (30% for poliovirus) and 52% in Luxembourg (Figure 2). The Netherlands targeted high-risk subpopulations, strategically focusing on areas with low vaccination coverage (for measles and poliovirus). Consequently, the representativeness of surveillance extended only to the sub-populations.

### Usefulness

In the post-pandemic scenario of 2023, no specific actions were exclusively triggered by WBS SARS-CoV-2 data, based on responses from participating countries. However, countries generally reported that WBS of SARS-CoV-2 was perceived as useful to make decisions of public health relevance, complementing information from other surveillance systems, e.g. clinical and hospital surveillance. Nine countries transmitted the results from their WBS systems to relevant authorities and public health actors on a weekly basis. Italy made WBS data available in real-time to the competent regional health authorities. Belgium, Denmark and Greece reported how respective authorities continuously assessed the needs and opportunities for the scale-up of their WBS systems. In general, the WBS results were discussed and evaluated internally at the institutes responsible for surveillance and with the relevant health authorities. Some countries, e.g. the Netherlands and Norway, collected feedback from end-users through surveys. Several countries highlighted the efficacy of WBS in monitoring the prevalence and trends of SARS-CoV-2 and its variants. Also, it was highlighted that the integration of WBS with other data sources, such as clinical surveillance, enhanced and made the monitoring of SARS-CoV-2 more comprehensive. In addition, WBS information was reported to be used to generate hypotheses for operational research, e.g. in Denmark, and to inform surveillance and preparedness strategies, e.g. in Norway. All countries communicated the WBS results to the public via dedicated websites and/or dashboards (Table 2). The majority of the countries (6/10) published their results weekly, while available dashboards were updated at different frequencies, e.g. daily in Austria, and on all working days in the Netherlands. Also, a few countries emphasised the importance of timely and effective communication of WBS findings. Public awareness efforts, such as the publication of results, were also recognised as essential factors in promoting transparency and community engagement.

The WBS systems of other pathogens than SARS-CoV-2 were mostly in their early stages, with some countries implementing pilot phases. Notable exceptions included the well-established poliovirus surveillance system in Finland, Italy and the Netherlands, where WBS was used to certify that the country is free of poliovirus circulation. In Finland, WBS also allowed the characterisation of other enteroviruses.

### Community participation

In none of the described WBS systems, the beneficiaries were reported to have a role or to influence defining the objectives, designing the representativeness and assessing the usefulness of the system in 2023. Finland, Hungary and Luxembourg remarked how their respective national media had been giving coverage to WBS surveillance, in view of its public interest. Overall, participants agreed on the importance to further discuss the role of communities in disease surveillance, e.g. via citizen science in the Netherlands.

## Discussion

We collected information about WBS systems of pathogens of human relevance in 10 European countries in 2023, a pivotal year to understand how WBS has advanced since the emergence of SARS-CoV-2. However, WBS systems are continuously adapting to the operational needs of respective countries and, with this study, we are looking at a snapshot of a developing surveillance tool.

WBS offers an opportunity for a high degree of representativeness of the population, because it covers all users of the wastewater system in a defined area. In the current post-pandemic scenario, SARS-CoV-2 WBS may offer the most reliable estimates on infection trends, as it does not depend on clinical testing [18]. Overall, the SARS-CoV-2 WBS systems of the countries participating in this study had a high population coverage (median: 46%) and samples were consistently taken over time, ensuring good representativeness.

However, it is worth considering here that representativeness may vary among countries based on geographical coverage and any given geographical coverage may not yield the same representativeness across different countries according to the distribution of the population. Moreover, most countries reported a geographical downscaling of their SARS-CoV-2 WBS systems towards mainly covering larger cities and high-density areas, potentially affecting the representativeness of the systems in regard to the inhabitants of sub-urban and peripheral areas [19]. Furthermore, it is important to interpret the representativeness and usefulness of WBS depending on the pathogen under surveillance. For instance, a countrywide 50% WBS coverage might be high enough to ensure good representativeness of SARS-CoV-2 circulation, given the relatively uniform risk of infection in the population. On the other hand, a pocket of low measles vaccination coverage, with it specifically high risk of infection, might not be captured by the same WBS system, depending on the coverage of the surveillance system in the given area.

Recently, the World Health Organization renewed its recommendations for WBS to complement public health surveillance [20]. All participants in this study reported that SARS-CoV-2 WBS was perceived as useful to make decisions of public health relevance. Although WBS data did not trigger any specific action in 2023, they complemented the results from other surveillance systems. It is also worth considering that, at this point in time, decisionmakers may not necessarily see WBS as an integral component of a public health surveillance system. With the exception of poliovirus surveillance, WBS is a relatively new instrument used for the surveillance of infectious diseases and its results need to be interpreted and translated in order to be meaningful and useful [1-4]. Decisionmakers might be more familiar with and trust more results from clinical testing, which is intuitively easier to interpret, i.e. the number of infected people in a population might be a more intuitive concept to communicate, understand and use than the concentration of viral copies per litre of wastewater. Therefore, a clear communication of the results, graphically, in writing and orally, is important to ensure the integration of WBS into the overall disease surveillance set up. This is crucial given the role that several actors in the European public health arena ascribe to WBS in revamping their preparedness strategies [21,22]. From a similar perspective, the involvement and participation of communities (the beneficiaries of surveillance) in designing tomorrow’s WBS systems will require an additional effort from both the implementers of the systems and the decisionmakers. For example, communities have shown different degrees of acceptance of WBS in regard to their privacy or the risk to stigmatise specific populations, while they aim to participate in the related decision-making process [23]. This participation would be also key to ensure that surveillance systems are perceived as and are actually relevant to their beneficiaries’ needs [24,25].

This study also documented the absence of established monitoring and evaluation processes of the WBS systems across countries. Although it is understandable how the urgent SARS-CoV-2 pandemic needs required and justified WBS systems to be run and developed in parallel [26], it is key that adequate monitoring and evaluation processes of WBS are now established in the post-pandemic scenario [16,27].

Additionally, we observed that various actors are often involved in national WBS, thus necessitating a high degree of collaboration between the different involved institutes, organisations, and their respective steering authorities, given the necessarily different scientific perspective of an institution aiming to academic research and that of one responsible for public health surveillance [28].

While not specifically enquired by the study questionnaire, no surveillance system was reported to routinely monitor/follow up WBS results from neighbouring countries for interpretation of country-specific results, although this could provide further insight on domestic findings. Hence, it remains unclear to what extent results from other European countries have been used by competent authorities in their respective countries. Initiatives like the Digital European Exchange Platform (EU4S-DEEP) have helped the mutual understanding of WBS systems across European countries and have offered opportunities for collaborations [5,7], while much remains to be done, e.g. in the standardisation and exchange of national/regional surveillance practices. This study lies on a collaboration across several European countries, which was possible thanks to the network of the EU-WISH project, which started in 2023. Given the revamped momentum around the WBS instrument in the wake of the SARS-CoV-2 pandemic, it is important that experiences, lessons learned, strengths and weaknesses of WBS systems are shared across countries and valued to advance in a more collaborative way.

Our study had some limitations. Firstly, this study included a selected group of countries with well-established WBS systems and that were actively participating in the network and in the design of the EU-WISH project at the time this study was conceived. Its findings therefore do not cover the diversity of practices, representativeness and usefulness of WBS systems in Europe, emphasising the need for caution in the generalisation of these results. Also, the findings of this study were based on information from individual experts who were involved in running and developing their respective national WBS systems. This might have resulted in biased evaluations; more detailed and broader analyses may address this concern in forthcoming studies. Secondly, the questionnaire used by this study was pre-tested by filling it in with Danish-related information and the answers were reviewed independently by two researchers. Thus, the questionnaire was not formally piloted and validated. However, the aim of this study was to describe the representativeness and usefulness of WBS in the post pandemic scenario and not to quantify the systems in their functional parts. Therefore, a validation of the study questionnaire was of relative less importance. Thirdly, this survey aimed to have an operational focus and to provide a timely overview of the use and applicability of the wastewater surveillance tool, at a juncture when the knowledge gained throughout the SARS-CoV-2 pandemic can be capitalised to address future threats. Thus, we did not investigate the diverse microbiological and epidemiological analysis methods of wastewater data, although they remain crucial aspects of the wastewater surveillance debate [29]. Exploring the methodological aspects of the related microbiological and epidemiological analysis methods across countries and pathogens would require another in-depth, specific survey to be adequately informative. Finally, the questionnaire’s scope was also limited because of its exclusive investigation of WBS systems of pathogens of human relevance. This decision primarily reflected the need to keep the analysis limited in content and time, while focusing on threats that are relevant to preparedness in the post-pandemic scenario, but it restricted the exploration of the broader possibilities of the WBS instrument. We should remark that this analysis did not explore and cover the multiple practices and uses that can be made of wastewater results, e.g. to monitor the presence of contaminants. Other experiences already in place, like the EU-WISH project, will now document and cover the possibilities of the WBS instrument in more detail.

We provided an overview of several European WBS systems, emphasising similarities and differences across the countries in terms of their representativeness and usefulness. In the process, we identified a number of questions that are key moving forward from the post-pandemic state into the pre-to-next-pandemic scenario (Box). We hope that these questions might trigger further analysis and debate on the WBS instrument and its role. Questions are addressed to both the research and decision-making communities.

Box **From the post-pandemic state into the pre-to-next-pandemic scenario – questions about the future of wastewater-based surveillance (WBS) in Europe.**

• What is the potential and relevance of WBS in the coming years in Europe and globally? What information should it provide to answer public health needs? What is its role alongside other surveillance systems and how can results be integrated with those of other surveillance systems?

• How can we foster international collaborations in WBS in light of future targets of interest?

• How can we ensure that results from WBS are easy to interpret and understand, and are being used for public health purposes?

## Conclusion

The reviewed WBS systems in 10 European countries had high population coverage, ensured good representativeness, and all countries reported that WBS of SARS-CoV-2 was perceived as useful to make decisions of public health relevance. However, more work is needed to ensure the integration of WBS into other surveillance systems in Europe, while WBS requires extensive interpretation to make it meaningful and useful for decisionmakers. Broader collaborations, like the newly established EU-WISH project, underlie the basis of the further development of WBS.

## Supplementary Data

499000 Supplement
