# Supplementary material for: A survey of the representativeness and usefulness of wastewater-based surveillance systems in 10 countries across Europe in 2023
Source: Euro Surveill. 2024 Aug 15;29(33):2400096. doi: 10.2807/1560-7917.ES.2024.29.33.2400096 (PMC11328501; doi:10.2807/1560-7917.ES.2024.29.33.2400096)
Supplement: Supplement [file 24-00096_BENEDETTI_Supplement.pdf]

Supplementary Materials – Representativeness and usefulness of wastewater-based surveillance systems in ten countries across Europe in 2023

Answers to the study questionnaire

This supplementary material is hosted by *Eurosurveillance* as supporting information alongside the article **Representativeness and usefulness of wastewater-based surveillance systems in ten countries across Europe in 2023**, on behalf of the authors, who remain responsible for the accuracy and appropriateness of the content. The same standards for ethics, copyright, attributions and permissions as for the article apply. Supplements are not edited by *Eurosurveillance* and the journal is not responsible for the maintenance of any links or email addresses provided therein.

This supplementary material includes the answers to the study questionnaire that informed the content of the article **Representativeness and usefulness of wastewater-based surveillance systems in ten countries across Europe in 2023**.

Answers by country

Austria	p. 2
Belgium	p. 6
Denmark	p. 11
Finland, a	p. 16
Finland, b	p. 21
Greece	p. 27
Hungary	p. 32
Italy	p. 37
Luxembourg	p. 42
The Netherlands	p. 47
Norway	p. 53

Answers to the study questionnaire

**Country – AUSTRIA**	
**Identification of the wastewater-based surveillance system(s)[1] in the country**	
Name of the system(s)	SARS-CoV-2 Abwassermonitoring in Österreich<br>(The Austrian SARS-CoV-2 wastewater-based surveillance system)
Relevant webpage	https://abwassermonitoring.at/ (only German)
Public, available documentation / information / literature about the wastewater-based surveillance system(s), with links	Description on the webpage (in German)
**Approach of the wastewater-based surveillance system(s) in the country**	
Pathogen(s) under surveillance in 2023	SARS-CoV-2 virus
Brief description of the system(s) in 2023, including the number of sampling sites, number of weekly samples, sampling methods and number of laboratories performing the analyses in 2023. Can also include information about e.g. sentinel surveillance, seasonal surveillance, *ad hoc* setup for emerging threats	In 2023, the system relies on 48 wastewater sampling sites, covering 58% of the population residing across all 9 Austrian provinces. For each sampling site, 2 weekly samples (24-hour composite flow-proportional samples) are collected and transferred to one central laboratory for analysis at Medical University Innsbruck/Institute of Legal Medicine (RT-PCR) and Medical University Vienna (variants determination)
Objectives of the system(s) in 2023	To monitor the national and regional SARS-CoV-2 situation in Austria (quantitative trends, qualitative – shifts in variants)
Use of non-wastewater data in the wastewater-based surveillance system(s)	Before 1.7.23: official notifications of human infections in surveillance regions have been used. Since then it is not a notifiable disease anymore. No additional data available

[1] System for the surveillance of human diseases

Sectors and actors implementing wastewater-based surveillance in 2023 (including their role in funding)	Public Health Sector (publicly funded), via different institutions under the Austrian Ministry of Social Affairs, Health, Care & Consumer Protections:<br>• Medical University Innsbruck/Institute of Legal Medicine (National Reference Laboratory)<br>• Medical University Vienna + Research Center for Molecular Medicine of the Austrian Academy of Sciences (CeMM-Austria)<br>• AGES-Austrian Agency for Health and Food Safety
Use case – if available, describe and/or reference how the implementers of the system(s) perform tasks (list of actions) and how they use the information generated by the system(s)	No specific use case – weekly reports for stakeholders
**Representativeness** – "A public health surveillance system that is representative accurately describes the occurrence of a health-related event over time and its distribution in the population by place and person", see here	
Population under surveillance in 2023: what is it and how is it identified?	58% of resident population in Austria (about 4.6 million inhabitants)<br>48 sampling sites: coverage of all regions – focus on larger cities
Describe and quantify the geographical coverage of the system(s) in 2023 e.g. regional/national; rural/urban	All 9 Austrian provinces<br>Focus on urban areas
Describe and quantify the frequency of the data collection in 2023	All 9 Austrian provinces<br>Focus on urban areas<br>Bi-weekly samples from each sampling site
Describe relevant infrastructural, legislative, financial or other matters that determine the coverage of the system(s) in 2023	EU Commission Recommendation 2021/472
Describe major changes in the representativeness of the system(s) since 2020	6/20 – 6/21: pilot research project<br>9/21 – 6/22: school location monitoring (122 sites)<br>1/22 – up to now: National Cov-2 monitoring: starting with 16 sites, enhanced to 32 in Dec. and 48 sites in 1/23

Considering the most relevant pathogen under wastewater surveillance today, how do you consider the system(s) to be representative of the population residing in the country? (tick one of the answers and comment your choice)	Pathogen: SARS-CoV-2<br><br>**Very representative X**<br>Somehow representative<br>Do not know / cannot say<br>Partially representative<br>Negligibly representative
**Usefulness** – "Usefulness implies that surveillance results are used for public health action. Assessing usefulness consists in taking inventory of actions that have been taken in conjunction with the surveillance system", see here	
Describe how the results of the system(s) are communicated in 2023 (internally/externally)	Weekly reports are disseminated and discussed internally to Ministry of Health and other relevant institutions. Wastewater data and results are also communicated to the public via the dedicated website.
Describe how the information gathered by the system(s) is utilized and for what purpose in 2023 e.g. detection of pathogens or other hazards; estimation of disease burden; detection of outbreaks; description of disease distribution, spread, trends, modality, risk factors; hypotheses to stimulate research; measuring results of control measures; guidance for public health planning	No concrete plans at the moment, but:<br>• Smaller national research project about the usage of wastewater surveillance for other pathogens (EU-WISH "light"<br>• Participation in EU-WISH
Describe the actors involved in utilizing the information gathered by the system(s) in 2023	See above

Provide example(s) of how public health actions are based on the information gathered by the system(s)	No actions, but continuous citation in public statements of minister; dramatic increase of web access of website since summer 2023
Describe how the actors implementing wastewater-based surveillance monitor how/if the surveillance system(s) is used for public health actions	System is used as an early warning system – since there was only a slight increase in summer 2023 no actions have been taken
Considering the most relevant pathogen under wastewater surveillance today, how do you consider the system(s) to be useful to make decisions of public health relevance? (tick one of the answers and comment your choice)	Pathogen: SARS-CoV-2<br><br>Very useful<br>**Somehow useful X**<br>Do not know / cannot say<br>Partially useful<br>Negligibly useful<br><br>The system gives an epidemiological perspective and shows trends. It does not help in outbreak detection or individual measures or for smaller regions.
**Role of the community (beneficiaries)**	
Describe the role of the community (beneficiaries of the surveillance activities) in defining the objectives, designing the representativeness and assessing the usefulness of the wastewater-based surveillance system(s) today	Today, the Austrian population has no direct influence on the Austrian wastewater surveillance system.

Answers to the study questionnaire

**Country – BELGIUM**	
**Identification of the wastewater-based surveillance system(s)[2] in the country**	
Name of the system(s)	*COVID-19 Wastewater surveillance*
Relevant webpage	Dashboard: Wastewater Covid-19 (sciensano.be) https://wastewater.sciensano.be/dashboard/covid19/en/<br>Webpage: National surveillance of SARS-CoV-2 and its variants in sewage. | sciensano.be https://www.sciensano.be/en/projects/national-surveillance-sars-cov-2-and-its-variants-sewage<br>Reports: WASTEWATER-BASED EPIDEMIOLOGICAL SURVEILLANCE OF THE SARS-COV-2 – WEEKLY REPORT – RESULTS OF 11/10/2023 (sciensano.be) https://covid-19.sciensano.be/sites/default/files/Covid19/COVID-19-Weekly_wastewater_surveillance.pdf
Public, available documentation / information / literature about the wastewater-based surveillance system(s), with links	Webpage: National surveillance of SARS-CoV-2 and its variants in sewage. | sciensano.be<br>https://www.sciensano.be/en/projects/national-surveillance-sars-cov-2-and-its-variants-sewage<br><br>SARS-CoV-2 Surveillance in Belgian Wastewaters<br>Janssens R, Hanoteaux S, Maloux H, Klamer S, Laisnez V, Verhaegen B, Linard C, Lahousse L, Delputte P, Terwagne M, Marescaux J, Pype R, Didy C, Dierick K, Van Hoorde K, Lesenfants M.<br>Viruses. 2022 Sep 2;14(9):1950. doi: 10.3390/v14091950.<br>PMID: 36146757<br>https://doi.org/10.3390/v14091950<br><br>Time series modelling for wastewater-based epidemiology of COVID-19: A nationwide study in 40 wastewater treatment plants of Belgium, February 2021 to June 2022<br>Bertels X, Hanoteaux S, Janssens R, Maloux H, Verhaegen B, Delputte P, Boogaerts T, van Nuijs ALN, Brogna D, Linard C, Marescaux J, Didy C, Pype R, Roosens NHC, Van Hoorde K, Lesenfants M, Lahousse L.<br>Sci Total Environ. 2023 Nov 15;899:165603. doi: 10.1016/j.scitotenv.2023.165603. Epub 2023 Jul 19.<br>PMID: 37474075<br>https://doi.org/10.1016/j.scitotenv.2023.165603
**Approach of the wastewater-based surveillance system(s) in the country**	
Pathogen(s) under surveillance in 2023	SARS-CoV-2 virus

[2] System for the surveillance of human diseases

Answers to the study questionnaire

Brief description of the system(s) in 2023, including the number of sampling sites, number of weekly samples, sampling methods and number of laboratories performing the analyses in 2023. Can also include information about e.g. sentinel surveillance, seasonal surveillance, *ad hoc* setup for emerging threats	In 2023, the surveillance system relies on 41 wastewater treatment plants and covers 42% of the Belgian population.<br>The wastewater samples collected twice per week on Mondays and Wednesdays from the influent of 41 wastewater treatment plants spread over Belgium and mainly in high population density area. Samples are collected over a period of 24 hours and analysed for the presence of SARS-CoV-2, the virus causing the COVID-19.<br>The evolution of the SARS-CoV-2 viral load measured over time is assessed through three alerting indicators: the High Circulation, the Fast Increase and the Increasing Trend indicators. These indicators are computed at different geographical levels: national, regional, provincial, and treatment plants.<br>The samples are analysed in 3 different laboratories, using the same protocol, but final data is homogenized by Sciensano
Objectives of the system(s) in 2023	To monitor the circulation of COVID-19 in the Belgian population.
Use of non-wastewater data in the wastewater-based surveillance system(s)	Data about new infections with SARS-COV-2 from other surveillance systems such as consultations at general practitioners, numbers of COVID-19 testing, molecular surveillance of SARS-CoV-2 and hospitalisations.<br>See respi-radar: respi_2023_41_nl.pdf (sciensano.be)<br>https://www.sciensano.be/sites/default/files/respi_2023_41_nl.pdf
Sectors and actors implementing wastewater-based surveillance in 2023 (including their role in funding)	• Sciensano (funded publicly by Fed Government and Regional Health Authorities)<br>• The Federal Government on Health, Food Chain Safety and Environment<br>• Regional Health Authorities (Zorg en Gezondheid, AVIQ, COCOM)
Use case – if available, describe and/or reference how the implementers of the system(s) perform tasks (list of actions) and how they use the information generated by the system(s)	No specific use case. See methods of the SARS-CoV-2 wastewater surveillance data in Belgium: COVID-19-Weekly_wastewater_surveillance-Annex_methodology.pdf (sciensano.be) https://covid-19.sciensano.be/sites/default/files/Covid19/COVID-19-Weekly_wastewater_surveillance-Annex_methodology.pdf
**Representativeness** – "A public health surveillance system that is representative accurately describes the occurrence of a health-related event over time and its distribution in the population by place and person", see here	
Population under surveillance in 2023: what is it and how is it identified?	• 42,3% of the resident population in Belgium (about 4.8 million inhabitants covered)<br>• The wastewater treatment plants (WWTPs) included in the surveillance were selected based on the following criteria:

	1. Plants covering areas with a high population density (> 400 inhab./km²) were selected as it was hypothesized that in such areas the potential for virus transmission is higher.<br>2. Plants covering a large population (> 50 000 inhab.) were selected in a cost efficiency approach.<br>3. At least two plants should be present in each province.<br>4. At least 30% of the population must be covered in each region<br>• Sampling sites are identified through a web application<br><br>See methods of the SARS-CoV-2 wastewater surveillance data in Belgium: COVID-19-Weekly_wastewater_surveillance-Annex_methodology.pdf (sciensano.be) https://covid-19.sciensano.be/sites/default/files/Covid19/COVID-19-Weekly_wastewater_surveillance-Annex_methodology.pdf
Describe and quantify the geographical coverage of the system(s) in 2023 e.g. regional/national; rural/urban	• All 10 Belgian provinces (3 regions) from which 206 municipalities are covered<br>• 41 wastewater treatment plants spread over Belgium and mainly in high population density area.<br>• Covered population by region: Folanders 36%, Brussels 99,5% and Wallonia 34,5%.<br>See methods of the SARS-CoV-2 wastewater surveillance data in Belgium: COVID-19-Weekly_wastewater_surveillance-Annex_methodology.pdf (sciensano.be) https://covid-19.sciensano.be/sites/default/files/Covid19/COVID-19-Weekly_wastewater_surveillance-Annex_methodology.pdf
Describe and quantify the frequency of the data collection in 2023	Twice a week, on Monday and Wednesday, samples are collected using 24 h flow-proportional auto-samplers.
Describe relevant infrastructural, legislative, financial or other matters that determine the coverage of the system(s) in 2023	Sciensano designed a model to ensure the representativeness of the system in monitoring trends and viral variants.
Describe major changes in the representativeness of the system(s) since 2020	From 2020 to June 2023, the system included 42 wastewater treatment plants and covered 45% of the Belgian population.<br>Since July 2023, the system was downscaled to 41 wastewater treatment plants (42,3% covered population), due to a closure of one of the wastewater treatment plants.
Considering the most relevant pathogen under wastewater surveillance today, how do you consider the system(s) to be representative of the	SARS-CoV-2<br><br>Very representative<br>**Somehow representative X**<br>Do not know / cannot say

population residing in the country? (tick one of the answers and comment your choice)	Partially representative<br>Negligibly representative
**Usefulness** – "Usefulness implies that surveillance results are used for public health action. Assessing usefulness consists in taking inventory of actions that have been taken in conjunction with the surveillance system", see here	
Describe how the results of the system(s) are communicated in 2023 (internally/externally)	Weekly, results are disseminated and discussed internally to Sciensano and other Health authorities. Wastewater data and results are also communicated to the public via the dedicated website and dashboard.<br>Weekly report: WASTEWATER-BASED EPIDEMIOLOGICAL SURVEILLANCE OF THE SARS-COV-2 – WEEKLY REPORT – RESULTS OF 11/10/2023 (sciensano.be) https://covid-19.sciensano.be/sites/default/files/Covid19/COVID-19-Weekly_wastewater_surveillance.pdf<br>Dashboard: Wastewater Covid-19 (sciensano.be) https://wastewater.sciensano.be/dashboard/covid19/en/
Describe how the information gathered by the system(s) is utilized and for what purpose in 2023 e.g. detection of pathogens or other hazards; estimation of disease burden; detection of outbreaks; description of disease distribution, spread, trends, modality, risk factors; hypotheses to stimulate research; measuring results of control measures; guidance for public health planning	In 2023, wastewater-based surveillance data are utilized to monitor the national and regional COVID-19 circulation in Belgium (jointly with other SARS-CoV-2 indicators in the framework of the national preparedness) and to develop hypotheses for operational research. Authorities continuously assess the needs and opportunities for the scaleup of the system.
Describe the actors involved in utilizing the information gathered by the system(s) in 2023	Sciensano, The Federal Government on Health, Food Chain Safety and Environment, regional Health authorities and universities.
Provide example(s) of how public health actions are based	Since its implementation, the system contributed to the design of the Belgian SARS-CoV-2 control strategy. Results are utilized jointly with other SARS-CoV-2 indicators in the framework of the national preparedness. Since May 2023 the test strategy in Belgium has been reconsidered and other SARS-COV-2 surveillance systems were

on the information gathered by the system(s)	stopped. At this moment the wastewater surveillance did became an important one in case of a rise of high circulation and in determining new variants of concern. In case of a warning on the level of wastewater surveillance a risk assessment group (that has his weekly meetings) will ask for our input and together there will be decided if health actions need to be taken.<br>Bulletin acute luchtweginfecties week 41-2023 | sciensano.be https://www.sciensano.be/en/biblio/bulletin-acute-luchtweginfecties-week-41-2023
Describe how the actors implementing wastewater-based surveillance monitor how/if the surveillance system(s) is used for public health actions	Passive feedback from The Federal Government on Health, Food Chain Safety and Environment, regional Health authorities and universities during a national risk assessment meeting.
Considering the most relevant pathogen under wastewater surveillance today, how do you consider the system(s) to be useful to make decisions of public health relevance? (tick one of the answers and comment your choice)	SARS-CoV-2<br><br>**Very useful X**<br>Somehow useful<br>Do not know / cannot say<br>Partially useful<br>Negligibly useful
**Role of the community (beneficiaries)**	
Describe the role of the community (beneficiaries of the surveillance activities) in defining the objectives, designing the representativeness and assessing the usefulness of the wastewater-based surveillance system(s) today	Today, the Belgian population has no direct influence on the Belgian wastewater surveillance system.

Belgium also implemented the following activities in 2023: RSV and Influenza in 3 WWTPs as a pilot study.

**Country – DENMARK**	
**Identification of the wastewater-based surveillance system(s)[3] in the country**	
Name of the system(s)	The Danish SARS-CoV-2 wastewater-based surveillance system
Relevant webpage	Webpage: https://www.ssi.dk/sygdomme-beredskab-og-forskning/sygdomsovervaagning/c/covid-19---spildevandsovervaagning (in Danish) https://en.ssi.dk/covid-19/national-surveillance-of-sars-cov-2-in-wastewater (in English)
Public, available documentation / information / literature about the wastewater-based surveillance system(s), with links	Description of the system available at: https://www.ssi.dk/-/media/cdn/files/covid19/spildevand---datagrundlag/datagrundlag-for-spildevandsovervaagning-af-sars-11102023.pdf (in Danish) https://en.ssi.dk/-/media/arkiv/uk/covid19/wastewater/data-description-for-the-wastewater-surveillance-of-sars-cov-2---11102023.pdf (in English)<br><br>McManus O, Christiansen LE, Nauta M, Krogsgaard LW, Bahrenscheer NS, von Kappelgaard L, Christiansen T, Hansen M, Hansen NC, Kähler J, Rasmussen A, Richter SR, Rasmussen LD, Franck KT, Ethelberg S. Predicting COVID-19 Incidence Using Wastewater Surveillance Data, Denmark, October 2021-June 2022. Emerg Infect Dis. 2023 Aug;29(8):1589-1597. doi: 10.3201/eid2908.221634. PMID: 37486168; PMCID: PMC10370843. Available at: https://www.ncbi.nlm.nih.gov/pmc/articles/PMC10370843
**Approach of the wastewater-based surveillance system(s) in the country**	
Pathogen(s) under surveillance in 2023	SARS-CoV-2 virus
Brief description of the system(s) in 2023, including the number of sampling sites, number of weekly samples, sampling methods and number of laboratories performing the analyses in 2023. Can also include information about e.g. sentinel surveillance, seasonal surveillance, *ad hoc* setup for emerging threats	In 2023, the system relies on 29 wastewater sampling sites, covering 47% of the population residing across the 5 Danish regions. For each sampling site, 2 weekly samples (24-hour composite flow-proportional samples) are collected and transferred to one central laboratory for analysis at Statens Serum Institut, the Danish national institute for infectious disease prevention and control. Analysis include viral detection and viral quantification (concentration). Trends of SARS-CoV-2 concentration (weekly average of the viral concentration and its growth rate) in wastewater are weekly monitored (nationally and regionally) in view of hospitalization data and other relevant indicators from individual testing.

[3] System for the surveillance of human diseases

Answers to the study questionnaire

Objectives of the system(s) in 2023	To monitor the national and regional SARS-CoV-2 infection rates in Denmark
Use of non-wastewater data in the wastewater-based surveillance system(s)	Data about new infections with SARS-CoV-2, the related hospitalizations (passive surveillance, based on national electronic registers) and SARS-CoV-2 sentinel surveillance are routinely used to interpret the trends of SARS-CoV-2 concentration in wastewater
Sectors and actors implementing wastewater-based surveillance in 2023 (including their role in funding)	Public Health Sector (publicly funded), via different institutions under the Danish Ministry of Health:<br>Statens Serum Institut<br>Danish Health Authority<br>Danish Patient Safety Authority<br><br>Wastewater treatment plans and the utility companies that manage the plants
Use case – if available, describe and/or reference how the implementers of the system(s) perform tasks (list of actions) and how they use the information generated by the system(s)	No specific use case. See the methods of the SARS-CoV-2 wastewater surveillance data in Denmark: https://www.ssi.dk/-/media/cdn/files/covid19/spildevand---datagrundlag/datagrundlag-for-spildevandsovervaagning-af-sars-11102023.pdf (in Danish) https://en.ssi.dk/-/media/arkiv/uk/covid19/wastewater/data-description-for-the-wastewater-surveillance-of-sars-cov-2---11102023.pdf (in English)
**Representativeness** – "A public health surveillance system that is representative accurately describes the occurrence of a health-related event over time and its distribution in the population by place and person", see here	
Population under surveillance in 2023: what is it and how is it identified?	46.5% of the resident population in Denmark (about 2.7 million inhabitants)<br>The 29 sampling sites (each with >30,000 inhabitants per catchment area) are chosen to cover at least 30% of the population in each of the 11 Danish provinces<br>Sampling sites are identified through a dedicated web application
Describe and quantify the geographical coverage of the system(s) in 2023 e.g. regional/national; rural/urban	All 11 Danish provinces (5 regions) are covered<br>Sampling sites privilege urban areas, where the largest shares of population reside<br>Covered population by region: The North Denmark Region47%, Central Denmark Region 40%, The Region of Southern Denmark 36%, The Capital Region of Denmark 68%, Region Zealand 32%
Describe and quantify the frequency of the data collection in 2023	Bi-weekly samples from each sampling site

Describe relevant infrastructural, legislative, financial or other matters that determine the coverage of the system(s) in 2023	Statens Serum Institut designed various models to ensure the representativeness of the system in monitoring trends and viral variants. Models were proposed to the Danish Ministry of Health, who chose their application based on surveillance objectives and resource availability (including the currently applied model)
Describe major changes in the representativeness of the system(s) since 2020	From July 2021 to June 2022, the system included up to 230 sampling sites and the largest covered resident population was 86.4% in December 2021. Then, the system was downscaled to 87 sampling sites (70% covered population) until February 2023. Since then, the system runs in the above-described setup.
Considering the most relevant pathogen under wastewater surveillance today, how do you consider the system(s) to be representative of the population residing in the country? (tick one of the answers and comment your choice)	Pathogen: SARS-CoV-2<br><br>Very representative<br>**Somehow representative X**<br>Do not know / cannot say<br>Partially representative<br>Negligibly representative
**Usefulness** – "Usefulness implies that surveillance results are used for public health action. Assessing usefulness consists in taking inventory of actions that have been taken in conjunction with the surveillance system", see here	
Describe how the results of the system(s) are communicated in 2023 (internally/externally)	Weekly, results are disseminated and discussed internally to Statens Serum Institut and other relevant institutions. Wastewater data and results are also communicated to the public via the dedicated website.<br>Relevant results are also published in Statens Serum Institut public reports about emerging risks – available at: https://www.ssi.dk/aktuelt/nyhedsbreve/nyt-fra-ssis-infektionsberedskab
Describe how the information gathered by the system(s) is utilized and for what purpose in 2023 e.g. detection of pathogens or other hazards; estimation of disease burden; detection of outbreaks; description of disease distribution, spread, trends, modality, risk factors;	In 2023, wastewater-based surveillance data are utilized to monitor the national and regional SARS-CoV-2 infection rates in Denmark (jointly with other SARS-CoV-2 indicators in the framework of the national preparedness) and to develop hypotheses for operational research. Authorities continuously assess the needs and opportunities for the scaleup of the system.

hypotheses to stimulate research; measuring results of control measures; guidance for public health planning	
Describe the actors involved in utilizing the information gathered by the system(s) in 2023	Statens Serum Institut and other relevant institutions under the Danish Ministry of Health
Provide example(s) of how public health actions are based on the information gathered by the system(s)	Since its implementation, the system contributed to the design of the Danish SARS-CoV-2 control strategy. Results are utilized jointly with other SARS-CoV-2 indicators in the framework of the national preparedness. In 2023, the information generated by the system did not translate into any public health action, yet.
Describe how the actors implementing wastewater-based surveillance monitor how/if the surveillance system(s) is used for public health actions	Passive feedback from the relevant institutions under the Danish Ministry of Health
Considering the most relevant pathogen under wastewater surveillance today, how do you consider the system(s) to be useful to make decisions of public health relevance? (tick one of the answers and comment your choice)	Pathogen: SARS-CoV-2<br><br>Very useful<br>**Somehow useful X**<br>Do not know / cannot say<br>Partially useful<br>Negligibly useful
**Role of the community (beneficiaries)**	
Describe the role of the community (beneficiaries of the surveillance activities) in defining the objectives, designing the representativeness and	Today, the Danish population has no direct influence on the Danish wastewater surveillance system.

assessing the usefulness of the wastewater-based surveillance system(s) today	

**Country – FINLAND, a**	
**Identification of the wastewater-based surveillance system(s)[4] in the country**	
Name of the system(s)	Environmental surveillance for poliovirus in Finland
Relevant webpage	https://thl.fi/fi/web/infektiotaudit-ja-rokotukset/taudit-ja-torjunta/taudit-ja-taudinaiheuttajat-a-o/polio/polion-jatevesiseuranta (in Finnish)
Public, available documentation / information / literature about the wastewater-based surveillance system(s), with links	Hovi T, Blomqvist S, Nohynek H, Savolainen-Kopra C. 2019. https://www.julkari.fi/bitstream/handle/10024/141597/SLL492019-2863.pdf?sequence=1&isAllowed=y. Article in Finnish, English abstract is available "Poliomyelitis is finally disappearing from the world – or is it?<br><br>Pitkänen T, Oikarinen S, Heikinheimo A, Hokajärvi AM, Blomqvist S, Savolainen-Kopra C. 2022. https://www.duodecimlehti.fi/duo16872 A review article of wastewater based surveillance in Finland. Article in Finnish.<br><br>Bubba, L.; Benschop, K.S.M.; Blomqvist, S.; Duizer, E.; Martin, J.; Shaw, A.G.; Bailly, J.-L.; Rasmussen, L.D.; Baicus, A.; Fischer, T.K.; et al. Wastewater Surveillance in Europe for Non-Polio Enteroviruses and Beyond. Microorganisms 2023, 11, 2496. https://doi.org/10.3390/microorganisms11102496
**Approach of the wastewater-based surveillance system(s) in the country**	
Pathogen(s) under surveillance in 2023	Poliovirus
Brief description of the system(s) in 2023, including the number of sampling sites, number of weekly samples, sampling methods and number of laboratories performing the analyses in 2023. Can also include information about e.g. sentinel surveillance, seasonal surveillance, *ad hoc* setup for emerging threats	In 2023, wastewater samples for poliovirus surveillance are collected from 5 sampling sites, covering about 30% of the Finnish population. Samples are collected from four WWTPs once a month and biweekly from one site with the largest population (Helsinki). The samples are 24-hour composite samples and they are transferred for analysis to one central laboratory, the national and regional reference laboratory for poliovirus, at the Finnish Institute for Health and Welfare. The poliovirus analysis is performed according to WHO guidelines for Environmental Polio Surveillance. Briefly, 500 ml of raw sewage is concentrated by 2-phase PEG-Dextran method and the concentrate is inoculated into cell cultures (RD and L20B). Cell cultures are observed by microscopy and all possible poliovirus isolates are further characterized by PCR methods.

[4] System for the surveillance of human diseases

Answers to the study questionnaire

Objectives of the system(s) in 2023	• To detect an emerging polio outbreak or any imported wild-type or PV2 or VDPV strains<br>• As a biproduct data is also gained on the circulation of other enteroviruses
Use of non-wastewater data in the wastewater-based surveillance system(s)	Data on polio vaccine coverage and laboratory results on any suspected polio (AFP) cases are gathered annually and reported to national authorities and WHO together with the data from the wastewater-based surveillance.
Sectors and actors implementing wastewater-based surveillance in 2023 (including their role in funding)	Public Health Sector (publicly funded)<br>• Ministry of Social Affairs and Health<br>• Finnish Institute for Health and Welfare under the Ministry of Social Affairs and Health<br>Wastewater treatment plants participate voluntarily.
Use case – if available, describe and/or reference how the implementers of the system(s) perform tasks (list of actions) and how they use the information generated by the system(s)	There is a regularly updated, publicly available National action plan for polio outbreak response in Finland. The Action plan describes the roles of different actors in case any wild-type, PV2 or VDPV strain is detected through wastewater based surveillance https://www.julkari.fi/bitstream/handle/10024/144284/URN_ISBN_978-952-343-870-5.pdf?sequence=1&isAllowed=y<br>The Action plan has been implemented once in 2009, when highly divergent VDPV poliovirus strains were detected in sewage samples in Tampere sewage network, Finland.<br>Roivainen M, Blomqvist S, Al-Hello H, Paananen A, Delpeyroux F, Kuusi M, Hovi T. Highly divergent neurovirulent vaccine-derived polioviruses of all three serotypes are recurrently detected in Finnish sewage. Euro Surveill. 2010 May 13;15(19):pii/19566. Erratum in: Euro Surveill. 2010;15(25). pii: 19594. Delpeyreux, F [corrected to Delpeyroux, F]. PMID: 20483108.<br>https://pubmed.ncbi.nlm.nih.gov/20483108/
**Representativeness** – "A public health surveillance system that is representative accurately describes the occurrence of a health-related event over time and its distribution in the population by place and person", see here	
Population under surveillance in 2023: what is it and how is it identified?	The current surveillance covers about 30% of the Finnish population. The selection of the sampling sites is based on the risk analysis made annually by the National polio laboratory in the Finnish Institute for Health and Welfare, and the National Certification Committee for poliovirus eradication, both working under the Ministry of Social Affairs and Health. The risk analysis is based on the current global progress in the eradication of polio. In 2023, the selected sampling sites represent a large target population, the main airports and harbours, the main target sites of refugees and the most favourite tourist destinations (Lapland).
Describe and quantify the geographical coverage of the system(s) in 2023 e.g. regional/national; rural/urban	Two of the sampling sites cover the Southern Capital region of Finland. Two sampling sites are in the largest cities of the Western Finland and one site is in the most popular tourist city in Lapland. Also, see the risk analysis described in the previous section.

Describe and quantify the frequency of the data collection in 2023	• Bi-weekly samples from 1 site<br>• Monthly samples from 4 sites
Describe relevant infrastructural, legislative, financial or other matters that determine the coverage of the system(s) in 2023	See above the risk analysis-based model for selecting the most relevant sampling sites.<br>Wastewater surveillance is not mentioned in the current Communicable Diseases Act and Decree. However, a large reform of the legislation has been initiated and this may change.
Describe major changes in the representativeness of the system(s) since 2020	There have not been major changes during recent years or even a decade. However, the number of sites sampled and the frequency of sampling may be changed if need be, e.g. depending on epidemiological situation in near areas.<br>The wastewater-based polio surveillance was started in Finland in 1960s and has since been continuously evaluated. For example, seasonal and geographical changes have been made according to the progress in the global eradication of polio. And in 2009, the sampling frequency was increased in one of the sampling sites due to the detections of VDPV strains.
Considering the most relevant pathogen under wastewater surveillance today, how do you consider the system(s) to be representative of the population residing in the country? (tick one of the answers and comment your choice)	Pathogen: Poliovirus<br><br>Very representative<br>**Somehow representative X**<br>Do not know / cannot say<br>Partially representative<br>Negligibly representative<br><br>We consider the current system representative enough. The risk for the emergence of polio outbreak in Finland is considered low.
**Usefulness** – "Usefulness implies that surveillance results are used for public health action. Assessing usefulness consists in taking inventory of actions that have been taken in conjunction with the surveillance system", see here	
Describe how the results of the system(s) are communicated in 2023 (internally/externally)	Wastewater-based polio surveillance is used to certify that Finland is free of poliovirus circulation. The results are reported annually to the National Certification Committee for poliovirus eradication, to the Ministry of Social Affairs and Health and to the European Regional Certification Committee.<br>In addition, all laboratory results are reported to WHO Global Polio Laboratory Network on a weekly basis.

	In case a programmatically relevant poliovirus strain is found, the communication will be according to the national Action Plan for polio outbreak response in Finland.
Describe how the information gathered by the system(s) is utilized and for what purpose in 2023 e.g. detection of pathogens or other hazards; estimation of disease burden; detection of outbreaks; description of disease distribution, spread, trends, modality, risk factors; hypotheses to stimulate research; measuring results of control measures; guidance for public health planning	In 2023, wastewater-based polio surveillance data are utilized to certify that Finland is free of polio circulation. Data gathered on circulation of other enteroviruses is also used to characterize type distribution of enteroviruses in Finland.
Describe the actors involved in utilizing the information gathered by the system(s) in 2023	The Finnish Institute for Health and Welfare, the Ministry of Social Affairs and Health, the National Certification Committee, the European Regional Certification Committee, the Global Polio Eradication Initiative coordinated by WHO
Provide example(s) of how public health actions are based on the information gathered by the system(s)	The public health actions depend on the characteristics of the detected poliovirus strain. Vaccine viruses do not induce any actions, and no other polioviruses have been detected since VDPV emergencies during 2009-2013.
Describe how the actors implementing wastewater-based surveillance monitor how/if the surveillance system(s) is used for public health actions	The actor implementing wastewater-based polio surveillance (the Finnish Institute for Health and Welfare) works under/in close collaboration with the Ministry of Social Affairs and Health. If any public health actions are needed, these will be planned and implemented together with these partners.

Considering the most relevant pathogen under wastewater surveillance today, how do you consider the system(s) to be useful to make decisions of public health relevance? (tick one of the answers and comment your choice)	Pathogen: poliovirus<br>**Very useful X**<br>Somehow useful<br>Do not know / cannot say<br>Partially useful<br>Negligibly useful<br><br>Wastewater-based poliovirus surveillance is the most important and only relevant tool for polio surveillance in Finland. The negative results are useful to certify that Finland is polio-free and new public health actions are not needed. The actions, which would follow the detection of any poliovirus with potential risk for public health, have been planned and agreed together with the decision makers.
**Role of the community (beneficiaries)**	
Describe the role of the community (beneficiaries of the surveillance activities) in defining the objectives, designing the representativeness and assessing the usefulness of the wastewater-based surveillance system(s) today	Today, the Finnish population has no direct influence on the Finnish wastewater surveillance system.

**Country – FINLAND, b**	
**Identification of the wastewater-based surveillance system(s)[5] in the country**	
Name of the system(s)	Wastewater-based surveillance of respiratory viruses and AMR in Finland
Relevant webpage	https://thl.fi/en/web/infectious-diseases-and-vaccinations/surveillance-and-registers/wastewater-monitoring
Public, available documentation / information / literature about the wastewater-based surveillance system(s), with links	SARS-CoV-2 public dashboard available at:<br>https://www.thl.fi/episeuranta/jatevesi/wastewater_weekly_report.html<br>For other pathogens, the information is provided in static webpages under the page:<br>https://thl.fi/en/web/infectious-diseases-and-vaccinations/surveillance-and-registers/wastewater-monitoring<br>For development of wastewater-based surveillance for pandemic preparedness, a project web page is available:<br>https://thl.fi/en/web/thlfi-en/research-and-development/research-and-projects/wastewater-based-surveillance-as-pandemic-preparedness-tool-wastpan-<br>The following scientific articles have been published:<br>Hokajärvi A-M, Rytkönen A, Tiwari A, Kauppinen A, Oikarinen S, Lehto K-M, Kankaanpää A, Gunnar T, Al-Hello H, Blomqvist S, Miettinen IT, Savolainen-Kopra C, Pitkänen T. (2021) The detection and stability of the SARS-CoV-2 RNA biomarkers in wastewater influent in Helsinki, Finland, Science of The Total Environment, 770, 145274, https://doi.org/10.1016/j.scitotenv.2021.145274<br><br>Tiwari A, Lipponen A, Hokajärvi A-M, Luomala O, Sarekoski A, Rytkönen A, Österlund P, Al-Hello H, Juutinen A, Miettinen IT, Savolainen-Kopra C, Pitkänen T. (2022) Detection and quantification of SARS-CoV-2 RNA in wastewater influent in relation to reported COVID-19 incidence in Finland. medRxiv 2021.10.05.21264462; doi: https://doi.org/10.1101/2021.10.05.21264462 and Water Research, 215, 118220, https://doi.org/10.1016/j.watres.2022.118220<br><br>Heljanko V, Johansson V, Räisänen K, Anttila V-J, Lyytikäinen O, Jalava J, Weijo I, Lehtinen J-M, Lehto K-M, Lipponen A, Oikarinen S, Pitkänen T, Heikinheimo A, and WastPan Study Group. (2023) Genomic epidemiology of nosocomial carbapenemase-producing Citrobacter freundii in sewerage systems in the Helsinki metropolitan area, Finland. Front Microbiol. doi: 10.3389/fmicb.2023.1165751.<br><br>Länsivaara AI, Lehto K-M, Hyder R, Janhonen E, Lipponen A, Heikinheimo A, Pitkänen T, Oikarinen A, WastPan study group (2023) Comparison of Different PCR Methods for the Detection of SARS-CoV-2 RNA in Wastewater

[5] System for the surveillance of human diseases

	Based on the Reported Incidence of COVID-19 in Finland. medRxiv 2023.09.07.23295183; doi: https://doi.org/10.1101/2023.09.07.23295183<br><br>Länsivaara A, Lehto K-M, Hyder R, Luomala O, Lipponen A, Hokajärvi A-M, Heikinheimo A, Pitkänen T, Oikarinen S, WastPan Study Group. (2023) Wastewater-based surveillance of respiratory syncytial virus epidemic at the national level in Finland. medRxiv 2023.09.04.23295011; doi: https://doi.org/10.1101/2023.09.04.23295011<br><br>Lehto K-M, Hyder R, Länsivaara A, Luomala O, Lipponen A, Hokajärvi A-M, Heikinheimo A, Pitkänen T, Oikarinen S, WastPan Study Group (2023) Wastewater-based surveillance is an efficient monitoring tool for tracking influenza A virus in the community. medRxiv 2023.08.28.23294723; doi: https://doi.org/10.1101/2023.08.28.23294723
**Approach of the wastewater-based surveillance system(s) in the country**	
Pathogen(s) under surveillance in 2023	SARS-CoV-2, Influenza A, Influenza B, RSV, and pilot scale: ESBL E. coli
Brief description of the system(s) in 2023, including the number of sampling sites, number of weekly samples, sampling methods and number of laboratories performing the analyses in 2023. Can also include information about e.g. sentinel surveillance, seasonal surveillance, *ad hoc* setup for emerging threats	The prevalence of coronavirus in untreated wastewater is reported weekly for the wastewater treatment plants in Espoo, Helsinki, Joensuu, Jyväskylä, Kuopio, Oulu, Tampere, Turku, Vaasa (n=9). Wastewater treatment plant in Rovaniemi and Lappeenranta are monitored once per month. The frequency of monitoring may be changed or other localities may be included in the monitoring if the epidemic situation so requires. The results are updated weekly on Fridays in the results report, in which the results of the coronavirus wastewater monitoring have been presented for each municipality. The results are presented in a weekly report on five different tabs: Entire Country, Localities Monitored Weekly, Localities Monitored Monthly, Monitoring History and Data. The first three tabs present results starting from 27 June 2022. The Monitoring History tab shows results for the period 3 August 2020 - 7 November 2022. The Data tab shows all the results starting from 3 August 2020. Wastewater samples in which coronavirus RNA is detected are sent for sequencing for coronavirus variants.<br>Starting from 31 July 2023 the Finnish wastewater-based respiratory virus surveillance was widened from coronavirus to include also weekly monitoring of influenza A, influenza B and RSV. In addition, as a pilot, the numbers of ESBL E. coli are monitored three times per year from the influent wastewater.<br>The samples are collected from the untreated wastewater inlet as a 24-hour composite sample. All analyses are carried out at the water microbiology laboratory of the Finnish Institute for Health and Welfare, Kuopio, Finland.
Objectives of the system(s) in 2023	Wastewater research offers an exceptional approach to assessing changes in the prevalence of infectious diseases on the population level. This type of research works best when repeated at regular intervals: samples are be used to observe city-specific changes in the prevalence of respiratory viruses, their variants, and selected multidrug resistant pathogens.

Use of non-wastewater data in the wastewater-based surveillance system(s)	In the SARS-COV-2 weekly report, the wastewater numbers are presented together with case numbers and hospitalization information. However, the SARS-CoV-2 variant data is presented on its own. Currently, the Finnish Institute for Health and Welfare is considering presenting the wastewater data in the future without the case information, since the individual testing rates are very low and thus not representative. The results of the clinical surveillance is presented separately.
Sectors and actors implementing wastewater-based surveillance in 2023 (including their role in funding)	The Finnish Institute for Health and Welfare (Governmental research institute under the Ministry of Social Affairs and Health, public health authority in Finland) conduct the surveillance on its own expenses.<br><br>Participating wastewater treatment plants take care of the sampling at their own expenses.
Use case – if available, describe and/or reference how the implementers of the system(s) perform tasks (list of actions) and how they use the information generated by the system(s)	The results are informed weekly to the epidemiologists and microbiologists responsible of infectious disease surveillance in national and regional levels in Finland. The results are complementary to other surveillance systems (clinical and sentinel surveillance systems, health care records and hospitalizations). The results can be used as early-warning to inform the local health care and to guide vaccination recommendations and timetable.
**Representativeness** – "A public health surveillance system that is representative accurately describes the occurrence of a health-related event over time and its distribution in the population by place and person", see here	
Population under surveillance in 2023: what is it and how is it identified?	Currently, approximately 44% of the wastewater produced by the Finnish population is covered by the wastewater monitoring. Existing sampling sites at the inlet of wastewater treatment plants are used. The site selection is dependent on the available resources and as wide as possible geographical coverage. The capital and the largest cities are included and also cities with known cross-border travelling.
Describe and quantify the geographical coverage of the system(s) in 2023 e.g. regional/national; rural/urban	A total of 11 cities are covered. Weekly sampling for the wastewater treatment plants in Espoo, Helsinki, Joensuu, Jyväskylä, Kuopio, Oulu, Tampere, Turku, Vaasa (n=9). Wastewater treatment plant in Rovaniemi and Lappeenranta are monitored once per month. The frequency of monitoring may be changed or other localities may be included in the monitoring if the epidemic situation so requires. In touristic season (winter months) sampling at Rovaniemi with high volume of international flights the sampling frequency is doubled.
Describe and quantify the frequency of the data collection in 2023	One sample per week (from Sunday to Monday morning). Once per month sampling for small part of the WWTPs.
Describe relevant infrastructural, legislative, financial or other matters that	The financial resources for wastewater surveillance need to be covered by the yearly budget for the institute. Therefore, prioritization and integration of different surveillance systems is needed (the work in progress).

determine the coverage of the system(s) in 2023	
Describe major changes in the representativeness of the system(s) since 2020	The number of sampling sites and the monitoring targets have been varied. While during these years, the number of samples has been decreased, the number of the monitoring parameters has increased.
Considering the most relevant pathogen under wastewater surveillance today, how do you consider the system(s) to be representative of the population residing in the country? (tick one of the answers and comment your choice)	Pathogen: SARS-CoV-2<br><br>**Very representative X**<br>Somehow representative<br>Do not know / cannot say<br>Partially representative<br>Negligibly representative<br><br>Comment: for the trends, wastewater surveillance is the only reliable data source. For the variants, the results align well with the results from the genetic surveillance of the clinical samples.<br><br>Pathogen: Other respiratory viruses (influenza A, influenza B, RSV)<br><br>Very representative<br>**Somehow representative X**<br>Do not know / cannot say<br>Partially representative<br>Negligibly representative<br><br>Comment: We are just starting with this surveillance and not yet knowledge about the normal levels and variation of the gene copy numbers. Still development needed to include the more detailed typing information of the circulating virus strains.<br><br>Pathogen: ESBL E. coli<br><br>Very representative<br>Somehow representative<br>**Do not know / cannot say X**

	Partially representative<br>Negligibly representative<br><br>Comment: At the moment, ESBL E. coli count information and strains are collected three times per year. However, there is further analysis nor reporting of these results yet available.
**Usefulness** – "Usefulness implies that surveillance results are used for public health action. Assessing usefulness consists in taking inventory of actions that have been taken in conjunction with the surveillance system", see here	
Describe how the results of the system(s) are communicated in 2023 (internally/externally)	The SARS-CoV-2 virus numbers are published weekly at the public website of the Finnish Institute for Health and Welfare and also distributed weekly to the national and regional health authorities responsible of communicable diseases by email. The coronavirus variant information is updated to the webpage on monthly basis. The results of the other respiratory viruses are currently distributed weekly internally and later on placed on the public web site too.<br><br>https://thl.fi/en/web/infectious-diseases-and-vaccinations/surveillance-and-registers/wastewater-monitoring
Describe how the information gathered by the system(s) is utilized and for what purpose in 2023 e.g. detection of pathogens or other hazards; estimation of disease burden; detection of outbreaks; description of disease distribution, spread, trends, modality, risk factors; hypotheses to stimulate research; measuring results of control measures; guidance for public health planning	Monitoring the trends of SARS-CoV-2 at each location monitored, and detection of the respiratory illness outbreaks
Describe the actors involved in utilizing the information gathered by the system(s) in 2023	The Finnish Institute for Health and Welfare under the Ministry of Social Affairs and Health, regional health authorities responsible of communicable diseases, general public, European Commission Joint Research Centre

Provide example(s) of how public health actions are based on the information gathered by the system(s)	Wastewater surveillance is a complementary source of data. The action is not needed, if the wastewater data is in agreement with the situational picture the health authorities have based on other indicators. In case there are any discrepancies, the public health action is to inform health care and thus increase awareness. The results might also affect the national level recommendations about the timing and coverage of the vaccination campaigns.
Describe how the actors implementing wastewater-based surveillance monitor how/if the surveillance system(s) is used for public health actions	The users of the wastewater data may leave feedback, questions and improvement proposals to the common email address jatevesiseuranta@thl.fi and also through the feedback system of the webpages. The wastewater surveillance system has been presented per request at courses and other gatherings of the users from the health care sector. Stakeholder discussion events have been arranged.
Considering the most relevant pathogen under wastewater surveillance today, how do you consider the system(s) to be useful to make decisions of public health relevance? (tick one of the answers and comment your choice)	Pathogen: Influenza A<br><br>Very useful<br>**Somehow useful X**<br>Do not know / cannot say<br>Partially useful<br>Negligibly useful<br><br>Comment: There is a need to start preparations for the next pandemic. Bird flu is one of the future concerns.
**Role of the community (beneficiaries)**	
Describe the role of the community (beneficiaries of the surveillance activities) in defining the objectives, designing the representativeness and assessing the usefulness of the wastewater-based surveillance system(s) today	The open web page with the possibility to download the actual surveillance data enables that anyone can make its own modelling and predictions. Such activity is often reported back to the jatevesiseuranta@thl.fi. Further, there has been media interest on this data source, and those discussions have been useful in understanding how the data should be presented in an enough simple way. The ease of interpretation helps also the health care professionals, not only public.

**Country – GREECE**	
**Identification of the wastewater-based surveillance system(s)[6] in the country**	
Name of the system(s)	National Wastewater Surveillance System (Greece)
Relevant webpage	https://eody.gov.gr/epidimiologika-statistika-dedomena/evdomadiaies-ektheseis/evdomadiaies-ektheseis-epidimiologikis-epitirisis-anapneystikon-loimoxeon/
Public, available documentation / information / literature about the wastewater-based surveillance system(s), with links	Koureas M; Amoutzias G; Vontas A; Kyritsi M; Pinaka O; Papakonstantinou A; Dadouli K; Hatziinikou M; Koutsolioutsou A; Mouchtouri V; Speletas M; Tsiodras S; Hadjichristodoulou C. Wastewater monitoring as a supplementary surveillance tool for capturing SARS-COV-2 community spread. A case study in two Greek municipalities. Environmental Research 2021;200 doi:10.1016/j.envres.2021.111749<br><br>Karapantsios T; Petala M; Kostoglou M; Dovas C; Lytras T; Paraskevis D; Roilides E; Koutsolioutsou-Benaki A; Panagiotakopoulos G; Sypsa V; Metallidis S; Papa A; Stylianidis E; Papadopoulos A; Tsiodras A; Papaioannou N. Relating SARS-CoV-2 shedding rate in wastewater to daily positive tests data: A consistent model-based approach. Science of the Total Environment, Volume 807, Part 2, 10 February 2022, Article 150838. https://doi.org/10.1016/j.scitotenv.2021.150838<br><br>Kostoglou M; Karapantsios T; Petala M; Roilides E; Dovas C; Papa A; Metallidis S; Stylianidis E; Lytras T, Paraskevis D; Koutsolioutsou-Benaki A; Panagiotakopoulos G; Tsiodras S; Papaioannou N. The COVID-19 pandemic as inspiration to reconsider epidemic models: A novel approach to spatially homogeneous epidemic spread modeling. Math Biosci Eng. 2022 Jul 11;19(10):9853-9876. doi: 10.3934/mbe.2022459
**Approach of the wastewater-based surveillance system(s) in the country**	
Pathogen(s) under surveillance in 2023	SARS-CoV-2 virus
Brief description of the system(s) in 2023, including the number of sampling sites, number of weekly samples, sampling methods and number of laboratories performing the analyses in 2023. Can also	In 2023, the system included 10 wastewater sampling sites, covering 50% of the population residing across 7 of the 13 Greek regions. For each sampling site, a minimum of 3 weekly samples (24-hour composite flow-proportional samples) were collected and transferred to six different laboratories (university or regional public health laboratories). Analysis includes concentration of the sample, viral RNA extraction, viral RNA quantification and wastewater viral load quantification. Trends of SARS-CoV-2 viral load (weekly average of the viral concentration) in wastewater are weekly monitored (nationally and regionally) vs. hospitalization data and other relevant indicators from individual testing.

[6] System for the surveillance of human diseases

include information about e.g. sentinel surveillance, seasonal surveillance, *ad hoc* setup for emerging threats	
Objectives of the system(s) in 2023	To monitor the SARS-CoV-2 circulation in Greece.
Use of non-wastewater data in the wastewater-based surveillance system(s)	Data about new infections with SARS-CoV-2, the related hospitalizations (passive surveillance, based on national electronic registers) and SARS-CoV-2 sentinel surveillance are routinely used to interpret the trends of SARS-CoV-2 concentration in wastewater.
Sectors and actors implementing wastewater-based surveillance in 2023 (including their role in funding)	Public Health Sector, publicly funded, under the Hellenic Ministry of Health. The National Wastewater Surveillance System is funded through the budget of the National Public Health Organisation.<br><br>Wastewater treatment plans and the utility companies that manage the plants.
Use case – if available, describe and/or reference how the implementers of the system(s) perform tasks (list of actions) and how they use the information generated by the system(s)	No specific use case.
**Representativeness** – "A public health surveillance system that is representative accurately describes the occurrence of a health-related event over time and its distribution in the population by place and person", see here	
Population under surveillance in 2023: what is it and how is it identified?	50% of the Greek population is monitored (about 5.2 million inhabitants)<br>The sampling sites were selected in order to cover the largest Greek cities, including all cities with population above 100,000 plus 3 cities with population below 100,000, which were included to expand the geographical scope of the network.
Describe and quantify the geographical coverage of the system(s) in 2023 e.g. regional/national; rural/urban	The sampling sites are located in 7 of the 13 Greek regions.<br>They cover urban populations.

Describe and quantify the frequency of the data collection in 2023	Minimum of three samples per week from each sampling site.
Describe relevant infrastructural, legislative, financial or other matters that determine the coverage of the system(s) in 2023	[missing]
Describe major changes in the representativeness of the system(s) since 2020	No major changes in the representativeness of the system have taken place since 2020. The National Wastewater Surveillance System started its operation in February 2021 with 9 sampling sites, which became 13 in summer 2021 and remained so until the end of 2022. In 2023, the final 10 sampling sites were selected based on the population and geographical scope covered. The per cent population covered did not change significantly, as the 3 sampling sites excluded in 2023 had small populations.<br>In 2021 and 2022, daily samples were collected from 2 sites. In 2023, daily samples were collected from 1 site (the sampling site in the Region of Attica, which is also the largest sampling site in terms of population covered).
Considering the most relevant pathogen under wastewater surveillance today, how do you consider the system(s) to be representative of the population residing in the country? (tick one of the answers and comment your choice)	Pathogen: SARS-CoV-2<br><br>Very representative<br>**Somehow representative X**<br>Do not know / cannot say<br>Partially representative<br>Negligibly representative
**Usefulness** – "Usefulness implies that surveillance results are used for public health action. Assessing usefulness consists in taking inventory of actions that have been taken in conjunction with the surveillance system", see here	
Describe how the results of the system(s) are communicated in 2023 (internally/externally)	Weekly. Results are provided to the National Public Health (NPHO) Organization by the collaborating laboratories, they are processed by a relevant algorithm and a report is produced. This report is incorporated in NPHO's weekly report about respiratory infections. The report is published on NPHO's website.
Describe how the information gathered by the system(s) is utilized and for what purpose	In 2023, wastewater-based surveillance data are used to monitor the circulation of the SARS-CoV-2 virus in the community, together with other surveillance data, such as the hospitalisations recorded and the data received from sentinel surveillance. The information gathered is used to assess the distribution of the disease, the burden

in 2023 e.g. detection of pathogens or other hazards; estimation of disease burden; detection of outbreaks; description of disease distribution, spread, trends, modality, risk factors; hypotheses to stimulate research; measuring results of control measures; guidance for public health planning	of the disease and the severity of the disease in comparison to previous years, as well as to guide public health planning. The information gathered is also used to develop hypotheses for research. Authorities continuously assess the needs and opportunities for the scale-up of the system.
Describe the actors involved in utilizing the information gathered by the system(s) in 2023	National Public Health Organization and the Public Health Committee of the Hellenic Ministry of Health.
Provide example(s) of how public health actions are based on the information gathered by the system(s)	Since its implementation, the system contributed to the design of the Danish SARS-CoV-2 control strategy. Results are utilized jointly with other SARS-CoV-2 indicators in the framework of the national routine surveillance and preparedness.
Describe how the actors implementing wastewater-based surveillance monitor how/if the surveillance system(s) is used for public health actions	The National Wastewater Surveillance Network has been established by the Public Health Sector itself and the results are obtained are utilised by it to better monitor the distribution of SARS-CoV-2 in the community.
Considering the most relevant pathogen under wastewater surveillance today, how do you consider the system(s) to be useful to make decisions of public health relevance? (tick one of the answers and comment your choice)	Pathogen: SARS-CoV-2<br><br>Very useful<br>**Somehow useful X**<br>Do not know / cannot say<br>Partially useful<br>Negligibly useful

**Role of the community (beneficiaries)**	
Describe the role of the community (beneficiaries of the surveillance activities) in defining the objectives, designing the representativeness and assessing the usefulness of the wastewater-based surveillance system(s) today	Today, the Greek population has no direct influence on the Danish wastewater surveillance system.

Greece also implemented the following activities in 2023: Other pathogens, e.g. Influenza A and B, RSV (Research efforts by National and Kapodistrian University of Athens, University of Thessaly, Aristotle University of Thessaloniki - not by NPHO).

**Country – HUNGARY**	
**Identification of the wastewater-based surveillance system(s)[7] in the country**	
Name of the system(s)	National Wastewater-based Surveillance System<br>Earlier: National COVID-19 early warning system
Relevant webpage	https://www.nnk.gov.hu/index.php/koronavirus/szennyvizvizsgalatok
Public, available documentation / information / literature about the wastewater-based surveillance system(s), with links	Description of the system available at:<br>https://www.nnk.gov.hu/index.php/kozegeszsegugyi-laboratoriumi-foosztaly/kornyezetegeszsegugyi-laboratoriumi-osztaly/vizhigienes-laboratorium/koronavirus-kimutatas-szennyvizbol-korai-elorejelzo-rendszer/szennyviz-koronavirus-monitorozas-kutatasi-jelentes (in Hungarian)<br>Scientific publications:<br>https://doi.org/10.1016/j.watres.2023.120098<br>https://doi.org/10.2166/wh.2022.179<br>https://doi.org/10.1016/j.scitotenv.2021.147398
**Approach of the wastewater-based surveillance system(s) in the country**	
Pathogen(s) under surveillance in 2023	SARS-CoV-2, Influenza A
Brief description of the system(s) in 2023, including the number of sampling sites, number of weekly samples, sampling methods and number of laboratories performing the analyses in 2023. Can also include information about e.g. sentinel surveillance, seasonal surveillance, *ad hoc* setup for emerging threats	The Hungarian wastewater-based surveillance system covers 23 weekly sampling sites: inlet of 3 WWTPs and collected sewage from the airport in the capital, Budapest; all county seats (18), and composite sample form 5 agglomeration cities near to Budapest. Approximately 40% of the population is monitored via the system. Sampling is carried out by the WWTP operators and transported by the public health authorities to the National Centre for Public Health and Pharmacy for sample processing and analysis. SARS-CoV-2 and Influenza A concentration are reported weekly. To the general public, trends and concentration categories of SARS-CoV-2 are published. SARS-CoV-2 variants from the largest cities are also identified by NGS if the SARS-CoV-2 concentration is sufficiently high.
Objectives of the system(s) in 2023	• Monitor COVID-19 trends nationally and regionally<br>• Monitor SARS-CoV-2 variants<br>• Monitor Influenza-A infection trends nationally

[7] System for the surveillance of human diseases

Use of non-wastewater data in the wastewater-based surveillance system(s)	Wastewater surveillance, clinical surveillance data and hospitalisation data feed into the same public health decision-making process. Variant data is also compared.
Sectors and actors implementing wastewater-based surveillance in 2023 (including their role in funding)	The National Center for Public Health and Pharmacy responsible for wastewater surveillance integrates many other relevant authorities, including the National Clinical Reference Laboratory (responsible for clinical respiratory surveillance), the National Epidemiological Authority and the National Healthcare Regulation Authority. These authorities are directly informed on the outcomes of the survey and contribute to designing further directions of the work.<br>Other involved partners:<br>WWTP operators<br>Local public health authorities
Use case – if available, describe and/or reference how the implementers of the system(s) perform tasks (list of actions) and how they use the information generated by the system(s)	No specific use case
**Representativeness** – "A public health surveillance system that is representative accurately describes the occurrence of a health-related event over time and its distribution in the population by place and person", see here	
Population under surveillance in 2023: what is it and how is it identified?	• 3 WWTP in the capital, Budapest<br>• Budapest Airport<br>• All county seats (n=18), which is usually the largest city in the county (>32,000 inhabitants)<br>• Composite sample form 5 agglomeration cities near to Budapest<br>• Approximately 40% of the population is monitored (3,9 million inhabitants)
Describe and quantify the geographical coverage of the system(s) in 2023 e.g. regional/national; rural/urban	• All county seats (18) and Budapest are monitored, thus the geographical coverage includes the entire country<br>• Sampling sites are all urban areas, where the largest share of population reside<br>• Population coverage by county varies between 8 and 45%, Budapest is covered 100%
Describe and quantify the frequency of the data collection in 2023	Weekly sampling from each sampling sites

Describe relevant infrastructural, legislative, financial or other matters that determine the coverage of the system(s) in 2023	Sampling scheme was designed based on the following considerations:<br>- at least one sample from every county to obtain full geographical coverage<br>- include every agglomeration above 150,000 inhabitants as per the EU Recommendation<br>- county seats were selected to utilise existing logistic networks<br>- cost/benefit analysis was conducted for increasing population coverage. It was found that to increase coverage from 40% to 50%, sample numbers should be increased by 150%. Taking into account the additional logistic burden, decision was to remain at 40% for the time being.
Describe major changes in the representativeness of the system(s) since 2020	Systematic weekly surveillance started in July 2020 with the Budapest WWTPs and county seats. Some agglomeration cities near Budapest are monitored since August 2020. From October 2021 to June 2023, two samples a week were taken from the biggest agglomerations (Budapest and 5 cities). Population coverage did not change significantly during the sampling period.
Considering the most relevant pathogen under wastewater surveillance today, how do you consider the system(s) to be representative of the population residing in the country? (tick one of the answers and comment your choice)	Pathogen: SARS-CoV-2<br><br>**Very representative X**<br>Somehow representative<br>Do not know / cannot say<br>Partially representative<br>Negligibly representative<br><br>Data analysis at the peak(s) of the pandemic indicated clear correlation (and before omicron, a distinct early warning capacity) for SARS-CoV2 morbidity and hospitalisation rates. In the current post-pandemic situation, when testing rates are declining and clinical reporting is limited, wastewater-based surveillance provides the most reliable estimates on infection trends.
**Usefulness** – "Usefulness implies that surveillance results are used for public health action. Assessing usefulness consists in taking inventory of actions that have been taken in conjunction with the surveillance system", see here	
Describe how the results of the system(s) are communicated in 2023 (internally/externally)	Official communication: SARS-CoV-2 and Influenza-A concentration trends at national and city-level are disseminated every week to the chief medical officer and to the other concerned departments (see Sectors and actors) in the National Centre for Public Health and Pharmacy. Wastewater surveillance data and clinical respiratory surveillance data are forwarded together to the Ministry of Interior, State Secretariat for Health.
Describe how the information gathered by the system(s) is utilized and for what purpose in 2023 e.g. detection of	In 2023, wastewater based surveillance data is used to support public health decision-making in combination with other relevant data such as clinical surveillance and hospitalisation rates on COVID-19 and influenza. The existence of such system also drives mapping additional potential use cases for WBS: current pilot is running on environmental surveillance for poliovirus (in a single WWTP in the capital).

pathogens or other hazards; estimation of disease burden; detection of outbreaks; description of disease distribution, spread, trends, modality, risk factors; hypotheses to stimulate research; measuring results of control measures; guidance for public health planning	
Describe the actors involved in utilizing the information gathered by the system(s) in 2023	National Center for Public Health and Pharmacy
Provide example(s) of how public health actions are based on the information gathered by the system(s)	During the pandemic, wastewater data was used for COVID-19 control measure decisions and preparedness of the healthcare system. Currently it is used to inform decision-makers but no action was deemed to be necessary in the post-pandemic period. It is also used for awareness raising through wide media coverage.
Describe how the actors implementing wastewater-based surveillance monitor how/if the surveillance system(s) is used for public health actions	See previous question
Considering the most relevant pathogen under wastewater surveillance today, how do you consider the system(s) to be useful to make decisions of public health relevance? (tick one of the answers and comment your choice)	Pathogen: SARS-CoV-2<br><br>Very useful<br>**Somehow useful X**<br>Do not know / cannot say<br>Partially useful<br>Negligibly useful

	It can be very useful if it is used in combination with other existing surveillance systems such as clinical or symptomatic surveillance.
**Role of the community (beneficiaries)**	
Describe the role of the community (beneficiaries of the surveillance activities) in defining the objectives, designing the representativeness and assessing the usefulness of the wastewater-based surveillance system(s) today	There is no direct feedback from the Hungarian population to the wastewater surveillance system. However, the SARS-CoV-2 wastewater result communication still receives great interest and wide media coverage in 2023. That means that the public still finds the system useful, and has a role in rise of public awareness.

Hungary also implemented the following activities in 2023: poliovirus (pilot).

**Country – ITALY**	
**Identification of the wastewater-based surveillance system(s)[8] in the country**	
Name of the system(s)	The Italian SARS-CoV-2 wastewater-based surveillance system
Relevant webpage	https://www.iss.it/cov19-acque-reflue
Public, available documentation / information / literature about the wastewater-based surveillance system(s), with links	[missing]
**Approach of the wastewater-based surveillance system(s) in the country**	
Pathogen(s) under surveillance in 2023	• SARS-CoV-2: systematically monitored.<br>• Influenza and SRV: pilot scheme planned for 2024.<br>• Other pathogens (e.g., Monkeypox, Adenovirus, Norovirus, Hepatitis E virus): non-systematically monitored as part of research activities.
Brief description of the system(s) in 2023, including the number of sampling sites, number of weekly samples, sampling methods and number of laboratories performing the analyses in 2023. Can also include information about e.g. sentinel surveillance, seasonal surveillance, *ad hoc* setup for emerging threats	In accordance with the EU Commission Recommendation 2021/472. The system encompasses 19/20 of the Italian regions and 2/2 ot the Autonomous Provinces, monitoring 167 wastewater treatment plants and serving an estimated 31.7 million population equivalents [this corresponds to an estimate 30% of the population, though an accurate calculation for all the national network is not possible]. The sampling scheme uses 24h composite samples and a sampling frequency of twice per week (urban centres above 150k inhabitants, as per Recc. 2021/472) or once/week (urban centres between 50k and 150k). As a result of this strategy, approximately 200 wastewater samples are collected on a weekly basis. Approximately 40 laboratories designated by the Regional competent authorities, including APPA/ARPA, IZS, universities and other institutions, oversee virus detection/quantification (PT samples provided by ISS for QA purposes). Variant analysis is performed on a monthly basis in a central laboratory (ISS). Since March 2023, with the suspension of funding, the system became voluntary with some regions decreasing the frequency or the number of sampling points. The system will be formally revised (number of sampling points and sampling frequency), following its planned inclusion in the Italian pandemic preparedness plan for respiratory pathogens.

[8] System for the surveillance of human diseases

Answers to the study questionnaire

Objectives of the system(s) in 2023	• To describe trend analysis of SARS-CoV-2 detection in urban wastewaters over time, as a descriptor of the dynamic of excretion of the virus in human faeces and, therefore, of the epidemiological trends in the population.<br>• To study of SARS-CoV-2 variants spread over time, is accomplished through regular monthly national “flash surveys”, systematically published on the ISS official website since October 2021.
Use of non-wastewater data in the wastewater-based surveillance system(s)	Data from other surveillance systems (primary genomic surveillance of SARS-CoV-2 variants) are used only for comparison purposes with variant analysis in wastewater (cross-check of agreement of the two surveillance systems).
Sectors and actors implementing wastewater-based surveillance in 2023 (including their role in funding)	Public Health Sector (publicly funded), under the Italian Ministry of Health. Additional fundings may be available at the Regionale/Province level form Regional Health authorities.<br>Research fundings (public/private) may also contribute to specific activities at the local level (e.g. grants for young scientists or for the study of specific aspects of ww surveillance).<br>Wastewater treatment plants and the utility companies that manage the plants participate on a voluntary basis or with specific cooperation agreements, with no compensation.
Use case – if available, describe and/or reference how the implementers of the system(s) perform tasks (list of actions) and how they use the information generated by the system(s)	The data generated by the system(s) are not currently used for decision-making.
**Representativeness** – “A public health surveillance system that is representative accurately describes the occurrence of a health-related event over time and its distribution in the population by place and person”, see here	
Population under surveillance in 2023: what is it and how is it identified?	The 167 sampling sites are chosen to cover centres with >50k inhabitants distributed over 20 of the 21 Italian Regions/Autonomous Provinces. An estimated coverage of 30% of the Italian population in is attributed to the systems (calculation based on population equivalents of the wastewater plants included in the surveillance and not on the population resident in the catchment areas).
Describe and quantify the geographical coverage of the system(s) in 2023 e.g. regional/national; rural/urban	• 20/21 Regions/Autonomous Provinces are covered<br>• Sampling sites are mainly associated to urban areas. The 167 WTPs being monitored cover centres with over 150k residents (n°=25) and centres with populations between 50k and 150k (n°=122). Moreover, due to their touristic relevance, additional sampling points (smaller municipalities with less than 50k inhabitants) were included in the surveillance based on the evaluation of Regions/A.P. (n°=20)

Describe and quantify the frequency of the data collection in 2023	The WTPs serving urban centres with more than 150k inhabitants are monitored twice per week as per Rec. 2021/472, while WTPs collecting wastewaters from centres with a population between 50k and 150k inhabitants are monitored once per week.
Describe relevant infrastructural, legislative, financial or other matters that determine the coverage of the system(s) in 2023	In agreement with EU Commission Recommendation 2021/472, the monitoring network includes Wastewater Treatment Plants (WTPs) located in all urban centres with more than 150k inhabitants. Based on the data of the Italian National Institute of Statistics (http://dati.istat.it/Index.aspx?QueryId=18460), only 25 urban centres with that size are present in Italy, accounting for approximately 11.5 million inhabitants (≈19% of the national population), distributed in 13 of the 21 Italian Regions/A.P. Considering the distribution of the Italian population in medium-size centres, urban centres with a population between 50k and 150k inhabitants were added to the sampling network to improve both the population and territorial coverage and the level of detail compared to the minimum requirements of Rec. 2021/472. According to the data of the Italian National Institute of Statistics, this led to the inclusion of additional 122 urban centres. Moreover, additional sampling points (smaller municipalities with less than 50k inhabitants) were included in the surveillance based on the evaluation of Regions/A.P., due to their touristic relevance.<br>The legal framework for system's implementation and the organization requirements were set in Law Decree n. 73 of 25.05.2021 and Ministry of Health/Ministry of Finances Decree n. 30.10.2021. Starting 2024, the
Describe major changes in the representativeness of the system(s) since 2020	Systematic sampling on the various WTPs was progressively activated by the Regions/Autonomous Provinces during the first months of surveillance, so the number of sampling points and samples examined per week has progressively increased since October 2021. The number of samples analysed weekly has doubled in 2022 compared to 2021 (from around 100 samples per week in 2021 to around 200 samples per week in 2022). Starting from April 2023, with the shift from a funded system to a voluntary one, the number of weekly samples has halved and is now at roughly 100 samples per week.
Considering the most relevant pathogen under wastewater surveillance today, how do you consider the system(s) to be representative of the population residing in the country? (tick one of the answers and comment your choice)	Pathogen: SARS-CoV-2<br><br>Very representative<br>**Somehow representative X**<br>Do not know / cannot say<br>Partially representative<br>Negligibly representative<br><br>Comment: The system was representative during the official surveillance period with dedicated funding from 1st October 2021 to 31st March 2023. However, with the transition to a voluntary system, it can only be partly

	considered representative. With the shift to a voluntary-based system the geographic representativeness was reduced; also reduction of sampling frequency negatively impacted trend analysis. Variant analysis scheme was substantially unaltered, with only a slight reduction in the number of samples (though slowed down by the increase of concurrent, non-SARS-CoV-2 related, activities)
**Usefulness** – "Usefulness implies that surveillance results are used for public health action. Assessing usefulness consists in taking inventory of actions that have been taken in conjunction with the surveillance system", see here	
Describe how the results of the system(s) are communicated in 2023 (internally/externally)	Wastewater data are available in real-time to the Regional/Province health competent authorities via the ARC-GIS central data collection system with customized access (i.e., each C.A. can access and visualize the data related to the corresponding territory). Access to all national data is granted to ISS (in charge of the ARC-GIS central data collection system) and to the national health competent authority (I.e., Ministry of Health).<br>Relevant results are periodically published in ISS website – available at: https://www.iss.it/cov19-acque-reflue
Describe how the information gathered by the system(s) is utilized and for what purpose in 2023 e.g. detection of pathogens or other hazards; estimation of disease burden; detection of outbreaks; description of disease distribution, spread, trends, modality, risk factors; hypotheses to stimulate research; measuring results of control measures; guidance for public health planning	Up to March 2023 (fully operative system) the data were used for SARS-CoV-2 trend analysis, new variants detection and analysis of their spread and to develop research hypothesis. Since April 2023 trend analysis at national level has been suspended due to reduction of system coverage (i.e., number of sampling points and frequency). Trend analysis has been maintained in some instances at regional/province level.
Describe the actors involved in utilizing the information gathered by the system(s) in 2023	Istituto Superiore di Sanità (ISS), the Italian Ministry of Health, the Regionale/Autonomous Provinces Health competent authorities.
Provide example(s) of how public health actions are based on the information gathered by the system(s)	Up to 2023, the information generated by the system were not translate into any public health action. Wastewater surveillance is considered a complementary source of data.

Describe how the actors implementing wastewater-based surveillance monitor how/if the surveillance system(s) is used for public health actions	Feedback from the regional/province or national health competent authorities.
Considering the most relevant pathogen under wastewater surveillance today, how do you consider the system(s) to be useful to make decisions of public health relevance? (tick one of the answers and comment your choice)	Pathogen: SARS-CoV-2<br><br>Very useful<br>**Somehow useful X**<br>Do not know / cannot say<br>Partially useful<br>Negligibly useful<br><br>Comment: To date the system has only been used as an additional information source
**Role of the community (beneficiaries)**	
Describe the role of the community (beneficiaries of the surveillance activities) in defining the objectives, designing the representativeness and assessing the usefulness of the wastewater-based surveillance system(s) today	The general population has no direct influence on the objectives, design or evaluation of the usefulness of the wastewater surveillance system.

Italy also maintained the following long-term activity in 2023: Environmental surveillance of poliovirus undertaken by the Regional Reference and Collaborating Centre for Polio (ISS - Dept. of Infectious Diseases) – poliovirus.

Country – LUXEMBOURG	
**Identification of the wastewater-based surveillance system(s)[9] in the country**	
Name of the system(s)	CORONASTEP
Relevant webpage	https://www.list.lu/en/covid-19/coronastep/
Public, available documentation / information / literature about the wastewater-based surveillance system(s), with links	https://www.list.lu/en/covid-19/coronastep/<br>https://publicationid.list.lu/coronastep-monitoring-of-sars-cov-2-in-luxembourg-wastewater
**Approach of the wastewater-based surveillance system(s) in the country**	
Pathogen(s) under surveillance in 2023	• SARS-CoV-2 virus and variants<br>• Seasonal human coronaviruses<br>• Influenza A and B<br>• RSV<br>• Norovirus (GGI and GGII)<br>• Human enterovirus
Brief description of the system(s) in 2023, including the number of sampling sites, number of weekly samples, sampling methods and number of laboratories performing the analyses in 2023. Can also include information about e.g. sentinel surveillance, seasonal surveillance, *ad hoc* setup for emerging threats	In 2023, the system includes 13 wastewater treatment plants covering 75% of the population.<br>For each sampling site: 1 weekly sample, 24-hour composite samples, fluxes of SARS-CoV-2 calculated using inlet water flows data. All samples are gathered for processing in one site; the Luxembourg Institute of Science and Technology which has been mandated to do the job by the Ministry of Health and the Ministry of Environment and Sustainable Development. Analyses include viral concentration step, viral RNA/DNA extraction, viral detection and quantification by RT-qPCR, variant detection by RT-ddPCR, data treatment, and reporting. SARS-CoV-2 wastewater data are weekly reported to health authorities. Trends of SARS-CoV-2 fluxes in wastewater are fortnightly publicly reported on our website. Data on other viruses of interest are not yet regularly reported to health authorities.

[9] System for the surveillance of human diseases

Objectives of the system(s) in 2023	• To monitor the national and regional SARS-CoV-2 (and its variants) infection rates in Luxembourg.<br>• Surveillance of other respiratory viruses in large<br>• An extension of the surveillance to AMR and chemicals is an objective for the end of 2023 – 2024.
Use of non-wastewater data in the wastewater-based surveillance system(s)	Luxembourg established rapidly after the onset of the pandemics in Europe a policy of large-scale PCR testing. This provided a very good picture of the prevalence and incidence of SARS-CoV-2 in Luxembourg. These data were made available for comparison with the dynamics of SARS-CoV-2 RNA fluxes in the monitored wastewater treatment plants. The correlation between the fluxes in wastewater and prevalence in the population has been established and used as a proof-of-concept of the usefulness of wastewater-based surveillance for managing the COVID-19 crisis. From April 2023, the large-scale direct PCR testing of the population ended and nowadays, the government is only relying on WBE data.
Sectors and actors implementing wastewater-based surveillance in 2023 (including their role in funding)	Public sector with<br>- Luxembourg Institute of Science and Technology, Research Institute of Public Utility<br>- Health Directorate, operational administration of the Ministry of Health<br>- Water Management Administration, belonging to the Ministry of Environment, Climate and Sustainable Development<br>- Wastewater treatment syndicates and municipalities
Use case – if available, describe and/or reference how the implementers of the system(s) perform tasks (list of actions) and how they use the information generated by the system(s)	No specific use case.
**Representativeness** – "A public health surveillance system that is representative accurately describes the occurrence of a health-related event over time and its distribution in the population by place and person", see here	
Population under surveillance in 2023: what is it and how is it identified?	• 75% of the populations = 495,667 inhabitants<br>• Inlet of 13 WWTPs is surveyed.<br>• The WWTPs have capacities between 5,000 and 210,000 inhabitant-equivalents.<br>• 6 sampling sites with >30,000 inhabitants per sewage catchment area
Describe and quantify the geographical coverage of the system(s) in 2023 e.g. regional/national; rural/urban	• The whole country is covered.<br>• All regions (urban and rural) are covered, while a higher =density of sampling is observed in the south of the country which is more urban and populated

Describe and quantify the frequency of the data collection in 2023	Weekly sampling for each sampling site
Describe relevant infrastructural, legislative, financial or other matters that determine the coverage of the system(s) in 2023	The coverage remains in 2023 as large as it was in 2022 thanks to a specific funding from Ministry of Health and Ministry of Environment, Climate and Sustainable Development. The reason for keeping a high coverage is the stopping of other surveillance tools such as the large-scale testing of the population by PCR on nasopharyngeal swabs. WBE is the last high-frequency running surveillance systems
Describe major changes in the representativeness of the system(s) since 2020	The surveillance started early in March 2020. A retrospective analysis using frozen samples has been made, providing evidence of a first circulation in Luxembourg in February 2020. Until July 2020, the surveillance network included 7 WWTPs representing 65% of the population. From August 2020 on, the network surveillance has been extended to 13 WWTPs. The frequency has been set to 2 or 3 sampling campaigns a week until June 2022. Then, the frequency has been set to one sample in each of the 13 monitored WWTPs per week. Discussions are in progress to downscale the number of WWTP while maintaining representativeness
Considering the most relevant pathogen under wastewater surveillance today, how do you consider the system(s) to be representative of the population residing in the country? (tick one of the answers and comment your choice)	Pathogen: SARS-CoV-2<br><br>Very representative<br>**Somehow representative X**<br>Do not know / cannot say<br>Partially representative<br>Negligibly representative<br>As a small country with a lot of cross-border workers from nearby countries, we are still working on the question of representativeness for the strictly resident population versus the neighbouring population, which makes up 212,000 of our total population of 660,809.
**Usefulness** – "Usefulness implies that surveillance results are used for public health action. Assessing usefulness consists in taking inventory of actions that have been taken in conjunction with the surveillance system", see here	
Describe how the results of the system(s) are	Once a week, results are sent to Ministry of Health / Health Directorate, Ministry of Environment, Climate and Sustainable Development / Water Management Administration, High Commission for National Protection. This

communicated in 2023 (internally/externally)	report is also made publicly available to the citizens on the following website twice a month.<br>https://www.list.lu/en/covid-19/coronastep/<br>A more specific website for all types of wastewater-based epidemiology will be available by the beginning of 2024.
Describe how the information gathered by the system(s) is utilized and for what purpose in 2023 e.g. detection of pathogens or other hazards; estimation of disease burden; detection of outbreaks; description of disease distribution, spread, trends, modality, risk factors; hypotheses to stimulate research; measuring results of control measures; guidance for public health planning	In Luxembourg, WBE data are used to<br>• monitor the regional and national levels of SARS-CoV-2 in the population<br>• to define circulation trends at the national and regional level<br>• to inform public health authorities<br>• to develop research questions<br>We currently work in close collaboration with health authorities to implement a permanent surveillance network for infectious pathogens.
Describe the actors involved in utilizing the information gathered by the system(s) in 2023	Luxembourg Institute of Science and Technology in charge of the surveillance send the information to<br>- Ministry of Health / Health Directorate<br>- Ministry of Environment, Climate and Sustainable Development / Water Management Administration<br>- High Commission for National Protection<br>- Prime Minister Office<br>The information sent week after week has been mentioned most often during the Prime Minister/Minister of Health press conferences
Provide example(s) of how public health actions are based on the information gathered by the system(s)	Since its implementation, the CORONASTEP data were officially included in the list of indicators (among clinical cases, hospitalisation…) followed by the health authorities to follow the pandemics trends and to deploy control strategy and restrictive measures.<br>Since the stop of systematic testing of the population, wastewater data are now one of the still remaining indicator to support public health authorities
Describe how the actors implementing wastewater-based surveillance monitor how/if the surveillance	Regular reporting of data is still ongoing once a week

system(s) is used for public health actions	
Considering the most relevant pathogen under wastewater surveillance today, how do you consider the system(s) to be useful to make decisions of public health relevance? (tick one of the answers and comment your choice)	SARS-CoV-2<br><br>**Very useful X**<br>Somehow useful<br>Do not know / cannot say<br>Partially useful<br>Negligibly useful<br><br>Comment: The Minister of Health has emphasised on the high usefulness of the WBE for the management of the crisis, from July 2020 on. This indicator has been included in the major indicators together with:<br>- Numbers of cases per day<br>- Reff (effective reproduction rate)<br>- Normal care hospital occupancy<br>- ICU occupancy<br>- Presence of Variants of concern
**Role of the community (beneficiaries)**	
Describe the role of the community (beneficiaries of the surveillance activities) in defining the objectives, designing the representativeness and assessing the usefulness of the wastewater-based surveillance system(s) today	It must be noted that the CORONASTEP data were very well received and followed by the country's population (high visit rate of the CORONASTEP page). CORONASTEP received very strong support from the local media, which relayed the data and encouraged the authorities to take them into account.

**Country – The Netherlands**	
**Identification of the wastewater-based surveillance system(s)[10] in the country**	
Name of the system(s)	The Dutch National Wastewater Surveillance program (NRS)
Relevant webpage	Open data sets:<br>- https://data.rivm.nl/meta/srv/dut/catalog.search#/metadata/a2960b68-9d3f-4dc3-9485-600570cd52b9<br><br>Explanation of data:<br>- https://coronadashboard.government.nl/verantwoording/virusdeeltjes-in-rioolwater<br><br>Coronavirus Dashboard (MoH):<br>- https://coronadashboard.government.nl/<br><br>RIVM webpage for national programme:<br>- https://www.rivm.nl/en/sewage-research/covid-19<br><br>RIVM Polio research webpage:<br>- https://www.rivm.nl/en/sewage-research/polio<br><br>RIVM AMR research webpage:<br>- https://www.rivm.nl/en/antimicrobial-resistance
Public, available documentation / information / literature about the wastewater-based surveillance system(s), with links	Open data & explanation: A list of publications and related documents can be found on:<br>https://www.rivm.nl/en/sewage-research/covid-19<br><br>as well as on:<br><br>https://coronadashboard.government.nl/verantwoording/virusdeeltjes-in-rioolwater<br><br>Publications:<br>The daily updated Dutch national database on COVID-19 epidemiology, vaccination and sewage surveillance

[10] System for the surveillance of human diseases

	E L P E Geubbels et al., Sci Data. 2023 Jul 20;10(1):469 https://www.nature.com/articles/s41597-023-02232-w<br><br>Patterns of SARS-CoV-2 circulation revealed by a nationwide sewage surveillance programme, the Netherlands, August 2020 to February 2022<br>M van Boven et al., Eurosurveillance, 2023, vol. 28, no. 25.<br>https://www.eurosurveillance.org/content/10.2807/1560-7917.ES.2023.28.25.2200700<br><br>The detection of monkeypox virus DNA in wastewater samples in the Netherlands<br>E.F. de Jonge et al., Science of The Total Environment, Vol. 852, 2022<br>https://www.sciencedirect.com/science/article/pii/S0048969722053645<br><br>Inferring Hospital Admissions from SARS-CoV-2 Virus Loads in Wastewater in the Netherlands, August 2020 – February 2022<br>https://www.sciencedirect.com/science/article/pii/S004896972307331X<br><br>Regional re-emergence of a SARS-CoV-2 Delta lineage amid an Omicron wave detected by wastewater sequencing<br>https://www.nature.com/articles/s41598-023-44500-0<br><br>SARS-CoV-2 in wastewater: potential health risk, but also data source<br>https://pubmed.ncbi.nlm.nih.gov/32246939/
**Approach of the wastewater-based surveillance system(s) in the country**	
Pathogen(s) under surveillance in 2023	• SARS-CoV-2<br>• Mpox virus (regular surveillance ended)<br>• Influenza virus (pilot)<br>• RS-virus (pilot)<br>• Measles virus (pilot)<br>• Polio virus (regular surveillance performed for several decades by RIVM by a different department through analysis of grab samples at strategic locations. https://www.rivm.nl/en/sewage-research/polio/)<br>• AMR (surveillance performed for several decades by RIVM by a different department through analysis at strategic locations) https://www.rivm.nl/en/sewage-research/antimicrobial-resistance

Answers to the study questionnaire

Brief description of the system(s) in 2023, including the number of sampling sites, number of weekly samples, sampling methods and number of laboratories performing the analyses in 2023. Can also include information about e.g. sentinel surveillance, seasonal surveillance, *ad hoc* setup for emerging threats	In 2023, all Dutch public WWTPs (311) are sampled, covering over 99% of the Dutch population. In January 2023, four samples per week were analysed (24-hour composite flow-proportional samples), in February 2024 three-, and from July 2023 onwards two per week.<br>All samples are transported via a cold-chain transport to, and analysed at the RIVM; SARS-CoV-2 quantification (all samples) and variant analysis (sequence analysis on a selection of samples) is performed.<br>Trends of viral loads are monitored, both on a national and on a regional level. Data obtained from the clinical surveillance, for instance positive tests, hospitalisations, are also used in comparison with the wastewater data.
Objectives of the system(s) in 2023	• To monitor SARS-CoV-2 circulation and trends among the Dutch population, wastewater surveillance on viral load (2x week all WWTPs) and variants (selection of ~70-96 locations per week)<br>• Influenza virus and RS-virus (pilots): wastewater surveillance on viral presence and quantifiable trends at 40 locations (Influenza A and B virus, RS-virus A and B), Influenza subtyping (H1 and H3) and possibly in the future; avian influenza detection.<br>• Mpox virus detection, if the epidemiological situation changes and more cases of mpox are found in the Netherlands.<br>• Inclusion of measles virus detection – pilot study.
Use of non-wastewater data in the wastewater-based surveillance system(s)	Data on the numbers of positive tests (when still available), the number of hospitalisations are used to compare wastewater data with other modes of COVID-19 surveillance.<br>Use the global virus variant circulation (clinical surveillance), such as VUM//VOC, to compare with WBE sequence data.
Sectors and actors implementing wastewater-based surveillance in 2023 (including their role in funding)	The Dutch National Wastewater Surveillance is funded by the Ministry of Health, Welfare and Sports (VWS).<br>The separate Waterboards (21), the associated Water laboratories, and the Association of Dutch Water Authorities (UvW) are included. The 21 Waterboards are responsible for 3-25 WWTPs each.
Use case – if available, describe and/or reference how the implementers of the system(s) perform tasks (list of actions) and how they use	The Outbreak Management Team (OMT) was an important advisor of the government during the Covid-19 crisis. In the advice of the 146th OMT COVID-19 meeting, wastewater was explicitly mentioned as showing that the severity of the impact of the virus on society was declining. We contributed to this advice with a graph, demonstrating the reduced disease burden of the virus on society. Our model shows a declining rate of hospitalisations at constant circulation of the virus in the population. Wastewater data was not used exclusively to

the information generated by the system(s)	come to the advice of the 146th OMT to stop the implementation of restrictive Covid-19 measures, but is was explicitly taken into account.
**Representativeness** – "A public health surveillance system that is representative accurately describes the occurrence of a health-related event over time and its distribution in the population by place and person", see here	
Population under surveillance in 2023: what is it and how is it identified?	>99% of the Dutch population (~ 17 million). This number is based on the registered residents of homes connected to the sewage systems via Statistics Netherlands. We do not exclude residents based on factors like age (small children may not use toilets, we do not take this into account (yet)).
Describe and quantify the geographical coverage of the system(s) in 2023 e.g. regional/national; rural/urban	National coverage, no distinction between any regions.
Describe and quantify the frequency of the data collection in 2023	• January - ~February: four-times a week all (311) WWTPs<br>• ~March - ~July: Three-times a week all WTTPs<br>• ~August-onwards: Two-times a week all WWTPs
Describe relevant infrastructural, legislative, financial or other matters that determine the coverage of the system(s) in 2023	The RIVM has written several memos for the MoH to describe the impact of downscaling from four-times a week all WWTPs are monitored to ultimately two-times a week all WWTPs. This to ensure that trends of viral loads/circulation are still sufficiently monitored and the wastewater surveillance program is more cost-effective.
Describe major changes in the representativeness of the system(s) since 2020	February 2020 started SARS-CoV-2 analysis: Schiphol Airport, two WWTPs, April 2020: 29 WWTPs, July 2020: 80 WWTPs, September 2020: All WWTPs (1x per week), July 2021: 2x per week, September 2021: 3x per week, November 2021: 4x per week.<br>2023, March 3x and August 2x per week.<br>Sequencing: selection.
Considering the most relevant pathogen under wastewater surveillance today, how do you consider the system(s) to be representative of the population residing in the country? (tick one of the answers and comment your choice)	SARS-CoV-2<br><br>**Very representative X**<br>Somehow representative<br>Do not know / cannot say<br>Partially representative<br>Negligibly representative

	Even with analysis 2x per week (all WWTPs) we can monitor both the regional as well as the national circulation of SARS-CoV-2 within the Dutch population.
**Usefulness** – "Usefulness implies that surveillance results are used for public health action. Assessing usefulness consists in taking inventory of actions that have been taken in conjunction with the surveillance system", see here	
Describe how the results of the system(s) are communicated in 2023 (internally/externally)	Each work day the wastewater viral loads are published as open data and published on the Coronavirus Dashboard (frequency of updating changed during 2023). Either weekly or later in 2023 bi-weekly reports on the SARS-CoV-2 wastewater data and explanation is shared with the MoH (confidential report) and the public https://www.rivm.nl/en/coronavirus-covid-19/weekly-figures
Describe how the information gathered by the system(s) is utilized and for what purpose in 2023 e.g. detection of pathogens or other hazards; estimation of disease burden; detection of outbreaks; description of disease distribution, spread, trends, modality, risk factors; hypotheses to stimulate research; measuring results of control measures; guidance for public health planning	WBE is used to monitor SARS-CoV-2 circulation in the Netherlands, together with the remaining clinical data. Other WBE pathogens are being investigated for potential inclusion, i.e. influenza virus, RS-virus, mpox virus, measles virus), as well as pilots for different life style parameters and chemicals (i.e. medicine residues, drugs, PFAS, …).
Describe the actors involved in utilizing the information gathered by the system(s) in 2023	SARS-CoV-2 data is published as open data and shown at the Coronavirus Dashboard. Other pathogens and substances/chemicals are communicated with the involved experts from the clinical and epidemiological surveillance, Municipal Health Services and the MoH.
Provide example(s) of how public health actions are based on the information gathered by the system(s)	Since its implementation, the system contributed to the national framework of monitoring the SARS-CoV-2 virus. Results are utilized jointly with other SARS-CoV-2 indicators in the framework of the national preparedness. In 2023, the information generated by the system did not translate into any public health action, yet.
Describe how the actors implementing wastewater-based surveillance monitor	A survey was performed among the Municipal Health Services if and how they used the wastewater surveillance data visualised at the Coronavirus Dashboard. Many MHSs replied that they frequently consulted the data to stay informed on trends in wastewater within their region.

how/if the surveillance system(s) is used for public health actions	
Considering the most relevant pathogen under wastewater surveillance today, how do you consider the system(s) to be useful to make decisions of public health relevance? (tick one of the answers and comment your choice)	SARS-CoV-2<br><br>**Very useful X**<br>Somehow useful<br>Do not know / cannot say<br>Partially useful<br>Negligibly useful<br><br>Because SARS-CoV-2 detection in wastewater is now one of the pillars in the surveillance of this virus in the Netherlands.
**Role of the community (beneficiaries)**	
Describe the role of the community (beneficiaries of the surveillance activities) in defining the objectives, designing the representativeness and assessing the usefulness of the wastewater-based surveillance system(s) today	Although the public is currently not directly involved in WBE we want to include citizens science in the future to investigate how different groups in society think about wastewater-based surveillance.

Country – NORWAY	
**Identification of the wastewater-based surveillance system(s)[11] in the country**	
Name of the system(s)	The Norwegian SARS-CoV-2 wastewater surveillance system (pilot)
Relevant webpage	Webpage: https://www.fhi.no/en/in/surveillance/wastewater-surveillance-of-infectious-diseases/results-from-wastewater-surveillance/
Public, available documentation / information / literature about the wastewater-based surveillance system(s), with links	Description of the system available at: https://www.fhi.no/en/in/surveillance/wastewater-surveillance-of-infectious-diseases/surveillance-of-SARS-CoV-2-in-wastewater-pilot-project/<br><br>Ettore A, Hyllestad S, Heradstveit P, Langlete P, Moen LV, Rohringer A, Pires J, Baz Lomba JA, Bragstad K, Feruglio SF, Aavitsland P, Madslien EH. Evaluation of the pilot wastewater surveillance for SARS-CoV-2 in Norway, June 2022 – March 2023. BMC Public Health 23, 1714 (2023). https://doi.org/10.1186/s12889-023-16627-2. Available at: https://link.springer.com/article/10.1186/s12889-023-16627-2
**Approach of the wastewater-based surveillance system(s) in the country**	
Pathogen(s) under surveillance in 2023	SARS-CoV-2 virus
Brief description of the system(s) in 2023, including the number of sampling sites, number of weekly samples, sampling methods and number of laboratories performing the analyses in 2023. Can also include information about e.g. sentinel surveillance, seasonal surveillance, *ad hoc* setup for emerging threats	In 2023, the system initially relied on 5 wastewater sampling sites, covering ~25% of the Norwegian population. It was downscaled in April to include 3 sampling sites, ~ 22% of the population. For each site, 2 weekly samples (24-72h composite flow-proportional) are collected and transferred to one contract laboratory located in Norway (Nemko Norlab).<br><br>The analysis performed by the contract laboratory include viral detection and quantification (concentration). Sequencing and variant analysis are performed at the Norwegian Institute of Public Health (NIPH).<br>Weekly trends of virus concentration and variant proportions are monitored by NIPH and compared with relevant clinical indicators.
Objectives of the system(s) in 2023	• Describe trends of SARS-CoV-2 virus circulation, including its variants in the population over time and space

[11] System for the surveillance of human diseases

	• Provide an early detection of change in infection trends in the population compared to other national COVID-19 surveillance systems and indicators.<br>• Detect and monitor emerging variants of public health relevance.
Use of non-wastewater data in the wastewater-based surveillance system(s)	The SARS-CoV-2 wastewater surveillance data are compared with data from the Emergency Preparedness Register for COVID-19 (Beredt C19) including the registration of all individual test results in the Norwegian Surveillance System for Communicable Diseases (MSIS) and national registries on intensive care unit (ICU) and hospitalisations.<br>https://www.fhi.no/en/id/corona/coronavirus/emergency-preparedness-register-for-covid-19/
Sectors and actors implementing wastewater-based surveillance in 2023 (including their role in funding)	Public health sector (NIPH) was responsible for the overall administration, coordination and funding of the project.<br><br>A network of reference contact persons was established for:<br><br>• the managers of enrolled wastewater treatment plants.<br>• the municipal doctors in participating municipalities (Oslo, Bergen, Trondheim, Tromsø, and Ullensaker.<br>• an outsourced private laboratory (contract laboratory) for the sample logistics and analysis of wastewater samples.
Use case – if available, describe and/or reference how the implementers of the system(s) perform tasks (list of actions) and how they use the information generated by the system(s)	No specific use case. See project information and the publication describing the evaluation of the system, available at https://www.fhi.no/en/in/surveillance/wastewater-surveillance-of-infectious-diseases/surveillance-of-SARS-CoV-2-in-wastewater-pilot-project/
**Representativeness** – "A public health surveillance system that is representative accurately describes the occurrence of a health-related event over time and its distribution in the population by place and person", see here	
Population under surveillance in 2023: what is it and how is it identified?	• 25% of the resident population in Norway (about 1.4 million inhabitants).<br>• The 5 sampling sites were chosen to represent the largest urban municipality in Norway as well as the airport with the highest international influx.<br>• Sampling sites were identified through dialogue with the relevant municipalities.
Describe and quantify the geographical coverage of the system(s) in 2023 e.g. regional/national; rural/urban	• In the period January-March 2023, 4 out of 11 Norwegian counties (3 out of 5 regions) were covered.<br>• From April 2023, 2 out of 11 counties were covered, representing the Eastern region of Norway, including the Oslo airport area and the urban area of the city of Oslo.

Describe and quantify the frequency of the data collection in 2023	• Bi-weekly samples from each sampling site.
Describe relevant infrastructural, legislative, financial or other matters that determine the coverage of the system(s) in 2023	The National Institute of Public Health (NIPH) chose their application and coverage based on national surveillance objectives/needs and resource/funding availability, which changed over time. Trend analysis estimates, considering different down-scale scenarios, were performed to guide the strategy of necessary downscaling. In these situations, national coverage was prioritized over regional representativeness.
Describe major changes in the representativeness of the system(s) since 2020	From June 2022 to November 2022, the system covered 30% of the Norwegian population (12 sampling sites; 5 municipalities). Due to budget restrictions, the system was downscaled December 2022 to include five sites (25% coverage). Then the system was further downscaled April 2023 to include three sites (22% coverage).
Considering the most relevant pathogen under wastewater surveillance today, how do you consider the system(s) to be representative of the population residing in the country? (tick one of the answers and comment your choice)	Pathogen: SARS-CoV-2<br><br>Very representative<br>Somehow representative<br>Do not know / cannot say<br>**Partially representative X**<br>Negligibly representative<br><br>After downscaling, the system lost most of its regional geographical representativeness (only 1 out of 5 regions/parts of the country included). However, the system still covers 22% of the population residing in the country. The ww trend-line corresponds well with the results from the clinical indicators at the national level.
**Usefulness** – "Usefulness implies that surveillance results are used for public health action. Assessing usefulness consists in taking inventory of actions that have been taken in conjunction with the surveillance system", see here	
Describe how the results of the system(s) are communicated in 2023 (internally/externally)	Weekly results are disseminated and discussed internally with the NIPH and relevant authorities within the health sector. Results are also disseminated and discussed with external stakeholders and published via the dedicated website (https://www.fhi.no/en/in/surveillance/wastewater-surveillance-of-infectious-diseases/). Relevant results

	are also reported and published in other NIPH surveillance reports (https://www.fhi.no/en/publ/2020/weekly-reports-for-coronavirus-og-covid-19/).
Describe how the information gathered by the system(s) is utilized and for what purpose in 2023 e.g. detection of pathogens or other hazards; estimation of disease burden; detection of outbreaks; description of disease distribution, spread, trends, modality, risk factors; hypotheses to stimulate research; measuring results of control measures; guidance for public health planning	Wastewater-based surveillance data are utilized to monitor national and local trends of SARS-CoV-2 infections and variants (together with other different indicators). Results are used to inform and guide public health authorities. Experiences are gathered to assess how wastewater-based data can be used in future preparedness against public health threats.
Describe the actors involved in utilizing the information gathered by the system(s) in 2023	Norwegian Institute of Public Health (NIPH) and other relevant institutions under the Norwegian Ministry of Health and Care Services.<br>Municipal doctors.
Provide example(s) of how public health actions are based on the information gathered by the system(s)	Results are utilized jointly with other indicators included in the national surveillance of SARS-CoV-2 under the responsibility of the National Institute of Public Health (NIPH). In 2023, the information did not translate into any public health action, but was used to inform and support national and local preparedness planning and surveillance.
Describe how the actors implementing wastewater-based surveillance monitor how/if the surveillance system(s) is used for public health actions	Through regular meetings and dialogue with relevant actors at the national and local level. In addition, the NIPH performed a survey in 2023, as part of the evaluation of the system, targeting the system's main stakeholders.<br>https://link.springer.com/article/10.1186/s12889-023-16627-2#Sec11

Considering the most relevant pathogen under wastewater surveillance today, how do you consider the system(s) to be useful to make decisions of public health relevance? (tick one of the answers and comment your choice)	Pathogen: SARS-CoV-2<br><br>Very useful<br>**Somehow useful X**<br>Do not know / cannot say<br>Partially useful<br>Negligibly useful<br><br>The system provides an early (1-2 weeks) signal of new waves of infection, compared to other indicators. It is resource-effective and independent of clinical testing and healthcare-seeking behaviour. The system can provide information about the circulation of new variants of concern before they are detected in patient samples. Temporary variations/fluctuations in viral load are sometimes causing challenges in result interpretation and communication.
**Role of the community (beneficiaries)**	
Describe the role of the community (beneficiaries of the surveillance activities) in defining the objectives, designing the representativeness and assessing the usefulness of the wastewater-based surveillance system(s) today	Today, the Norwegian population has no direct influence on the Norwegian wastewater surveillance system
